# Ionic Liquid Forms of the Antimalarial Lumefantrine in Combination with LFCS Type IIIB Lipid-Based Formulations Preferentially Increase Lipid Solubility, In Vitro Solubilization Behavior and In Vivo Exposure

**DOI:** 10.3390/pharmaceutics12010017

**Published:** 2019-12-22

**Authors:** Erin Tay, Tri-Hung Nguyen, Leigh Ford, Hywel D. Williams, Hassan Benameur, Peter J. Scammells, Christopher J. H. Porter

**Affiliations:** 1 Medicinal Chemistry, Monash Institute of Pharmaceutical Sciences, Monash University, 381 Royal Parade, Parkville, Victoria 3052, Australia; Erin.tay@monash.edu (E.T.); leigh.ford@lonza.com (L.F.); 2 Drug Delivery, Disposition and Dynamics, Monash Institute of Pharmaceutical Sciences, Monash University, 381 Royal Parade, Parkville, Victoria 3052, Australia; tri-hung.nguyen@monash.edu; 3 Oral Drug Delivery Innovation, Lonza Pharma Biotech & Nutrition, Monash Institute of Pharmaceutical Sciences, Monash University, 381 Royal Parade, Parkville, Victoria 3052, Australia; Hywel.williams@lonza.com; 4 Oral Drug Delivery Innovation, Lonza Pharma Biotech & Nutrition, 67412 Strasbourg, France; hassan.benameur@lonza.com; 5 ARC Centre of Excellence in Convergent Bio-Nano Science and Technology, Monash University, 381 Royal Parade, Parkville, Victoria 3052, Australia

**Keywords:** ionic liquid, lipophilic salt, drug delivery, drug absorption, poorly water-soluble drug, lumefantrine, lipid-based formulation, SEDDS, lipid formulation classification system

## Abstract

Lipid based formulations (LBFs) are commonly employed to enhance the absorption of highly lipophilic, poorly water-soluble drugs. However, the utility of LBFs can be limited by low drug solubility in the formulation. Isolation of ionizable drugs as low melting, lipophilic salts or ionic liquids (ILs) provides one means to enhance drug solubility in LBFs. However, whether different ILs benefit from formulation in different LBFs is largely unknown. In the current studies, lumefantrine was isolated as a number of different lipophilic salt/ionic liquid forms and performance was assessed after formulation in a range of LBFs. The solubility of lumefantrine in LBF was enhanced 2- to 80-fold by isolation as the lumefantrine docusate IL when compared to lumefantrine free base. The increase in drug loading subsequently enhanced concentrations in the aqueous phase of model intestinal fluids during in vitro dispersion and digestion testing of the LBF. To assess in vivo performance, the systemic exposure of lumefantrine docusate after administration in Type II-MCF, IIIB-MCF, IIIB-LCF, and IV formulations was evaluated after oral administration to rats. In vivo exposure was compared to control lipid and aqueous suspension formulations of lumefantrine free base. Lumefantrine docusate in the Type IIIB-LCF showed significantly higher plasma exposure compared to all other formulations (up to 35-fold higher). The data suggest that isolation of a lipid-soluble IL, coupled with an appropriate formulation, is a viable means to increase drug dose in an oral formulation and to enhance exposure of lumefantrine in vivo.

## 1. Introduction

Many currently marketed drugs, and drugs in development, are poorly water-soluble and classified as class II or class IV as defined by the Biopharmaceutics Classification System (BCS) [1,2,3,4]. Class II compounds are poorly water-soluble, but have high membrane permeability, while class IV compounds have both poor water solubility and poor permeability. Reformulation approaches are commonly employed to improve the bioavailability of class II compounds; however, for class IV compounds, the challenge is significantly greater and whilst improvements are possible via formulation approaches, it is often more beneficial to develop alternative analogues with more suitable physicochemical properties [5].

The increasing prevalence of poorly water-soluble drugs has necessitated the development of a range of formulation approaches to increase apparent drug solubility in the gastro-intestinal fluids [6,7,8,9]. These include the use of solid dispersions, lipid-based formulations, cyclodextrins, surfactants, and particle size reduction techniques [7]. The current studies have focused on the use of lipid-based formulations in conjunction with alternate lipophilic salt forms (or ionic liquids) to promote drug solubility in lipid-based formulations. The anti-malarial drug, lumefantrine, has been employed as a model poorly water-soluble drug. Lumefantrine is currently formulated as a tablet and is dosed in combination with artemether [10]. The oral bioavailability of lumefantrine is 4–11% [11]. The low and likely solubility limited bioavailability of lumefantrine may be improved by administration with a fatty meal, but this leads to variable bioavailability [11].

Ionic liquids (ILs) are generally defined as organic salts with melting points below 100 °C [12,13,14]. They are usually composed of an organic cation and an inorganic/organic anion [15,16]. Inefficient crystal packing of the ions and/or a more diffuse charge on either or both ionic species leads to the weakening of the interactions between the cation and anion in ILs. Using a bulky or irregularly shaped counterion further decreases inter-molecular interactions and the efficiency of crystal packing, leading to a decreased melting point [1,17,18]. The reduction in melting point observed in ILs typically increases solubility in both aqueous and non-aqueous vehicles, but has been employed here to enhance solubility in non-aqueous LBF vehicles.

Active pharmaceutical ingredient-ionic liquids (API-ILs) comprise a drug and an appropriate counterion. For the formation of an IL, there are a wide range of cation-anion combinations available and previous studies have shown that different API-ILs can result in improvements in drug solubility, stability, bioavailability, and membrane permeability [1,19,20,21,22,23]. The flexibility of counterion choice enables selection of an appropriate counterion for specific drug delivery systems. Here, we focus on lipophilic counterions to increase the solubility of ILs in lipid-based formulations.

Lipid-based formulations (LBFs) are composed of combinations of traditional lipids (such as monoglycerides, diglycerides, and triglycerides), surfactants, and co-solvents [24,25]. LBFs are commonly used as vehicles for poorly water-soluble drugs as they increase drug solubilization in the gastro-intestinal (GI) tract via integration into endogenous lipid solubilization pathways. As the drug is usually pre-dissolved in the formulation, LBFs also avoid traditional solid–liquid dissolution in the GI tract, a process that is often the rate-limiting step in absorption [26,27,28].

The solubilization capacity of LBFs, however, typically changes as the formulation is digested and interacts with bile salt micelles in the GI tract, leading to changes in structure [5]. Drug solubilization is more efficient at high lipid loads, especially in the presence of medium-chain lipids. These medium-chain lipids are digested very rapidly and efficiently, and at high concentration, promote drug solubilization [27,29]. However, at lower lipid loads, medium-chain lipids can lead to drug precipitation and reduced absorption as they are digested to form medium-chain fatty acids. Medium-chain fatty acids are relatively polar, and at low concentrations, swell bile salt micelles relatively poorly (thereby reducing overall solubilization capacity). In contrast, long-chain lipid digestion products are less polar, swell bile salt micelles more effectively, and typically lead to more robust drug solubilization, even at low lipid concentrations [28].

In 2006, Pouton developed a general system for the classification of lipid-based formulations (the Lipid Formulation Classification System (LFCS)). The LFCS aids in identifying the critical performance characteristics of lipid systems [5]. Type I formulations require digestion and are composed of only oils. Type II formulations contain lipids and water-insoluble surfactants and self-emulsify on contact with the GI fluids (i.e., self-emulsifying drug delivery systems (SEDDS)) [30,31,32]. Type III formulations are self-microemulsifying drug delivery systems (SMEDDS) or self-nanoemulsifying drug delivery systems (SNEDDS) and are sub-classified into Type IIIA and Type IIIB. Type IIIA formulations contain lipids, water-soluble surfactants and/or co-solvents, and Type IIIB contain similar components but have greater proportions of water-soluble surfactants and co-solvents than Type IIIA. Type IV formulations contain only hydrophilic surfactants and co-solvents [5].

A potential limitation of the utility of LBFs is low drug loading in the formulation such that the therapeutic drug dose cannot be solubilized in a volume of lipid that can be filled into one or two capsules [6]. The use of ionic liquid forms of drugs can increase drug loading in LBFs [6,32,33,34], but whether solubilization is maintained at these higher drug loads as the formulation is dispersed and digested is not well understood. Similarly, the relationship between formulation type and composition and the fate of an API-IL during formulation digestion has not been widely explored. The aim of the current study was to explore the ability of IL technology to promote lumefantrine solubility in range of LFCS class formulations, to explore the ability of the high load LBF to maintain drug in a solubilized state during dispersion and digestion, and ultimately to evaluate whether this translates into improved systemic exposure after oral administration. Recent interest in the ability of LBF to promote drug supersaturation in the GI tract also promoted an analysis as to whether the high drug loads that IL formation facilitate also allow for the facile generation of highly supersaturated conditions in the GI tract.

## 2. Materials and Methods

**Materials.** Lumefantrine was purchased from AK Scientific (Union City, CA, USA). Dioctyl sulfosuccinate sodium salt (sodium docusate) was purchased from Sigma Aldrich (St. Louis, MO, USA). Sodium dodecyl sulfate was purchased from BASF (Castle Hill, NSW, Australia). Hydrogen chloride in diethyl ether (2.0 M) was obtained from Sigma-Aldrich (St. Louis, MO, USA). Chloroform, methanol, and dichloromethane were purchased from Merck (Melbourne, VIC, Australia). Details of the LBFs used in the study are provided in Table 1. Captex^®^ 355 EP/NF and Capmul^®^ MCM EP were supplied by Anzchem (Sydney, NSW, Australia). Tween™ 85 was supplied by Croda (Sydney, NSW, Australia). Kolliphor^®^ RH 40 was obtained from BASF (Melbourne, VIC, Australia). Maisine™ 35-1 was supplied by Trapeze Associates Pty. Ltd. (Sydney, NSW, Australia). Soybean oil and butylated hydroxytoluene (BHT) were purchased from Sigma Aldrich (St. Louis, MO, USA). Ethanol was purchased from Merck (Melbourne, VIC, Australia). Sodium taurodeoxycholate > 95% (NaTDC), 4-bromophenylboronic acid, and porcine pancreatin (8 X USP specification activity) were purchased from Sigma Aldrich (St. Louis, MO, USA). Phosphatidylcholine (PC) (Lipoid E PC S, ~99.2% pure, from egg yolk) was obtained from Lipoid (Ludwigshafen, Germany). The 0.6 M and 0.2 M strength sodium hydroxide solutions were diluted from a stock solution of 1.0 M sodium hydroxide that was purchased from Science Supply (Melbourne, VIC, Australia). Formic acid was purchased from Sigma Aldrich (Sydney, NSW, Australia) and acetonitrile, both HPLC and LC-MS grade, was purchased from Merck (Melbourne, VIC, Australia). All solvents used were of analytical purity or high-performance liquid chromatography (HPLC) grade.

**Lumefantrine Hydrochloride Preparation.** Lumefantrine (3 g, 5.66 mmol) was dissolved in anhydrous dichloromethane (50 mL) and a solution of 2.0 M hydrochloride in diethyl ether was added dropwise to the solution (2.85 mL, 5.66 mmol). The solution was stirred for 3 h at room temperature. Dichloromethane was then evaporated and the residual solid product was dried under vacuum. (Mass: 3.02 g, Yield: 94%).

**Ionic Liquid Preparation: Lumefantrine Dodecyl Sulfate.** Lumefantrine hydrochloride (1.5 g, 2.65 mmol) was dissolved in methanol (200 mL) and sodium dodecyl sulfate (0.77 g, 2.65 mmol) was added. The solution was stirred for 3 h at room temperature. The methanol was then evaporated in vacuo and the residue was dried under vacuum overnight. The residue was treated with chloroform (200 mL) to dissolve the IL and precipitate out the sodium chloride. The mixture was filtered through a 2 µm micro filter, the solvent evaporated, and the final solid product dried under vacuum. (Mass: 1.76 g, Yield: 83%).

**Ionic Liquid Preparation: Lumefantrine Docusate.** Lumefantrine hydrochloride (1.5 g, 2.65 mmol) was dissolved in methanol (200 mL) and dioctylsulfosuccinate sodium salt (docusate) (1.2 g, 2.65 mmol) was added. The solution was stirred for 3 h at room temperature. The methanol was then evaporated in vacuo and the residue was dried under vacuum overnight. The residue was treated with chloroform (200 mL) to dissolve the IL and precipitate out the sodium chloride. The mixture was filtered through a 2 µm micro filter, the solvent evaporated, and the final semi-solid product dried under vacuum. (Mass: 1.96 g, Yield: 77%).

**Nuclear Magnetic Resonance (NMR) Spectroscopy.**^1^H and ^13^C NMR spectra were obtained at 400.13 Hz and 100.62 Hz respectively, on a Bruker Advance III Nanobay 400 MHz spectrometer coupled to the BACS 60 automatic sample changer. All spectra were processed using MestReNova 6.0 software. The chemical shifts of all ^1^H signals were measured relative to the expected solvent peaks of the NMR solvent; 2.50 ppm (DMSO-*d*_6_). The data for all spectra are reported in the following format: chemical shift (integration, coupling constant J (Hz), multiplicity). Multiplicity is defined as; s = singlet, d = doublet, t = triplet, q = quartet, dd = doublet of doublets, dt = doublet of triplets, and m = multiplet. Subsequent abbreviations also include J (Hz) = coupling constant in Hertz.

The ^1^H and ^13^C NMR spectra and MS data for lumefantrine hydrochloride, lumefantrine dodecyl sulfate, and lumefantrine docusate are provided in the supporting information.

**Liquid Chromatography-Mass Spectroscopy (LC-MS).** LC-MS chromatograms were obtained using an Agilent 6100 Series Single Quad LC/MS coupled with an Agilent 1200 Series HPLC, 1200 Series G1311A quaternary pump, 1200 series G1329A thermostatted autosampler, and 1200 series G1314B variable wavelength detector. The conditions for liquid chromatography were: reverse phase HPLC analysis using a Phenomenex Luna C_8_(2) 5 μm (50 × 4.6 mm) 100 Å column at a temperature of 30 °C. 5 μL of sample was injected and the sample was run in solvent A of 99.9% acetonitrile, 0.1% formic acid with a gradient of 5–100% (*v*/*v*) solvent A over 10 min. Solvent B was 99.9% water with 0.1% formic acid. Detection was at a UV wavelength of 254 nm. The conditions for mass spectrometry were: quadrupole ion source with multimode-ES, drying gas temperature 300 °C, and vaporizer temperature 200 °C. The capillary voltage was 2000 V in positive mode, or 4000 V in negative mode and the scan range was 100–1000 *m*/*z* with a step size of 0.1 s over 10 min.

**High-Resolution Mass Spectrometry.** All high-resolution mass spectrometry analyses were performed on an Agilent 6224 TOF LC/MS Mass Spectrometer coupled to an Agilent 1290 Infinity HPLC (Agilent, Palo Alto, CA, USA). All data were acquired and reference mass corrected via a dual-spray electrospray ionization (ESI) source. Each scan or data point on the Total Ion Chromatogram (TIC) is an average of 13,700 transients, producing a spectrum every second. Mass spectra were created by averaging the scans across each peak and background subtracting against the first 10 s of the TIC. Acquisition was performed using the Agilent Mass Hunter Data Acquisition software version B.05.00 Build 5.0.5042.2 and analysis was performed using Mass Hunter Qualitative Analysis version B.05.00 Build 5.0.519.13. The MS conditions were: electrospray ionization, a drying gas flow of 11 L/min at a temperature of 325 °C, a nebulizer at 45 psi, a capillary voltage of 4000 V, the fragmentor at 160 V, the skimmer at 65 V, and the OCT RFV of 750 V. The scan range acquired was 100–1500 *m*/*z*. The internal reference ions in positive ion mode had a *m*/*z* of 121.0509 and 922.0098. Chromatographic separation was performed using an Agilent Zorbax SB-C18 Rapid Resolution HT 2.1 × 50 mm, 1.8 µm column (Agilent Technologies, Palo Alto, CA, USA) using an acetonitrile gradient (5% to 100%) over 3.5 min at 0.5 mL/min.

**Polarized Light Microscopy and Hot Stage Microscopy.** The melting point ranges of all compounds were assessed using a hot stage microscope on an Axiolab Laboratory Microscope (manufactured 1997, S/N 982650) supplied by Carl Zeiss (Carl Zeiss, Oberkochen, Germany). The microscope was fitted with cross polarizing filters and coupled to a Linkam HFS91 hot stage connected to a Linkam TP93 system controller (Linkam Scientific Instruments, Tadworth, UK). Samples, mounted between two glass coverslips, were heated at 5° C/min until the compound showed signs of melting, at which time the rate was decreased to 1° C/min, and this was monitored continuously. Images were captured with a Canon LA-DC52C PowerShot A70 camera at 10 × magnification using Canon Utilities RemoteCapture version 2.7.2.16 software. Complete melting was defined as the lowest temperature at which the sample was free of birefringence; in the case of the amorphous sample (lumefantrine docusate), complete melting was defined as the lowest temperature where no solid structures were evident and therefore might also be described as a solid–liquid transition temperature.

**Equilibrium Solubility Studies.** The equilibrium solubility of lumefantrine, lumefantrine hydrochloride, lumefantrine dodecyl sulfate, and lumefantrine docusate in each formulation was determined by initially adding 20 mg of compound to 200 mg of LBF in a microcentrifuge tube. Each equilibrium solubility experiment was completed in triplicate. Equilibrium solubility was defined when solubility measured across two consecutive days varied less than 5%. The samples were allowed to equilibrate at 37 °C and were vortex-mixed twice a day to ensure the compounds were well dispersed in the LBF. The samples were left to equilibrate for at least 3 days. If all the compound was observed to be fully dissolved, the sample was centrifuged for 15 min at 14,800 rpm (21,000× *g*) at 37 °C (Thermo Scientific, Heraeus Pico 21 Centrifuge, Langenselbold, Germany) to confirm complete dissolution. Where no pellet was observed, another 20 mg of compound was added, and the process was repeated until complete dissolution was no longer observed and excess IL was evident. At each time point, samples were then centrifuged at 37 °C (14,800 rpm (21,000× *g*), Thermo Scientific, Heraeus Pico 21 Centrifuge) and aliquots of 20 mg were taken from the supernatant and dissolved in 1 mL of chloroform: methanol (2:1, *v*/*v*). The chloroform: methanol solution was then diluted 20-fold with water: acetonitrile (1:1, *v*/*v*). The samples were then assayed by HPLC to determine the concentration of compound in formulation.

**In Vitro Drug Solubilization.** The LBF were assayed using previously reported in vitro dispersion and digestion tests in order to assess the potential for formulations containing dissolved API-IL to maintain drug in a solubilized state as the formulation is dispersed and digested under simulated gastro-intestinal conditions [35]. Drug loading in each formulation was at 80% of the equilibrium solubility of the drug or API-IL in that formulation to maintain a consistent thermodynamic activity across all LBFs.

In vitro dispersion and digestion experiments were conducted using a pH-stat apparatus (Metrohm^®^ AG, Herisau, Switzerland), which comprised a Titrando 802 propeller stirrer/804 Ti Stand combination, a glass pH electrode (iUnitrode), and two 800 Dosino dosing units coupled to 10 mL autoburettes (Metrohm^®^ AG). The pH-stat was connected to a PC and operated via Tiamo 2.0 software (Metrohm^®^) [35]. First, 1.100 g of formulation was dispersed in 40 mL of bile salt/phospholipid micelles in simulated intestinal fluid (2 mM tris-maleate, 150 mM NaCl, 1.4 mM CaCl_2_·2H_2_O, 3 mM NaTDC, 0.75 mM PC, pH 6.5, 37 °C) and then 4 mL of pancreatic enzyme extract (pancreatin) was added to stimulate digestion. The pancreatic enzyme was prepared by mixing 1 g of porcine pancreatin with 5 mL of lipolysis buffer and 20 µL of 5 M NaOH. After mixing, this was then centrifuged at 2880× *g* at 5 °C for 10 min (Crown Scientific, Eppendorf AG Centrifuge 5804 R, Sydney Australia) and the supernatant used as the enzyme solution. The lipolysis buffer was prepared by dissolving 0.474 g of tris maleate, 0.206 g of CaCl_2_·H_2_O, and 8.775 g of NaCl in one liter of distilled water to form a 2 mM tris maleate 1.4 mM CaCl_2_·H_2_O 150 mM NaCl buffer; the pH was adjusted to 6.5 using NaOH. The micellar solution was prepared by dissolving 0.783 g of NaTDC and 0.291 g of PC in 500 mL of lipolysis buffer to form a 3 mM NaTDC 0.75 mM PC solution. The temperature-controlled vessel was held at 37 °C, and contained bile salt/phospholipid micellar solution in simulated intestinal fluid.

After dispersion of the formulation, digestion was initiated by addition of the pancreatic lipase/co-lipase solution. This resulted in the liberation of fatty acids (FAs) and therefore a drop in pH. This drop in pH was detected by the pH-stat controller, which then titrated the FA produced using an autoburette that added NaOH to keep the pH at a set point (0.6 M NaOH for MCFs, and 0.2 M NaOH for LCFs). By knowing the quantity of NaOH added and assuming stoichiometric titration, this indirectly quantified the extent of FA production (as a measure of digestion). At time intervals of −10, −5, and 0 (i.e., in the 15 min dispersion phase), and 5, 10, 15, 30, and 60 min (digestion phase—i.e., after initiation of digestion at time = 0), 1 mL aliquots were taken. Samples taken during digestion were immediately treated with a lipolysis inhibitor (10 µL of 0.5 M 4-bromophenylboronic acid in methanol per 1 mL of digestion medium) to halt digestion. All samples were then centrifuged to form a maximum of 3 phases—an oil phase, an aqueous phase, and a pellet phase.

**Figure 1:**
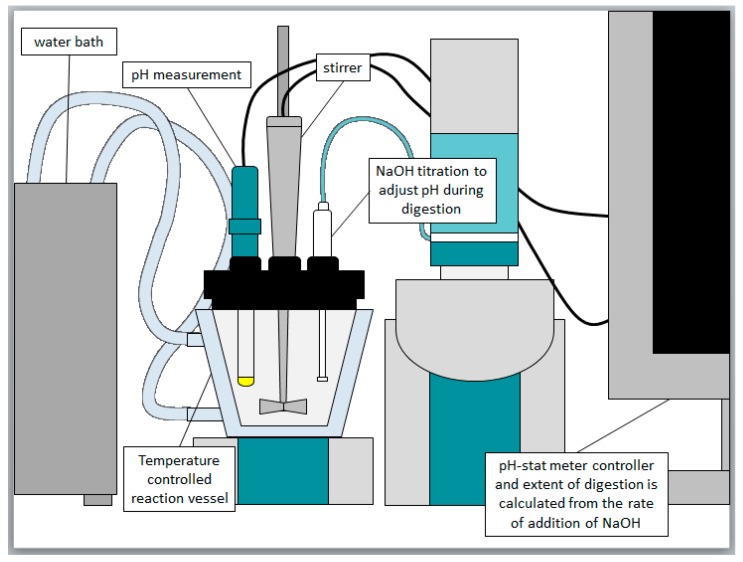
In vitro digestion model for testing lipid formulations. A temperature controlled (37 °C) vessel containing digestion buffer, bile salt, and phospholipid is used. The lipid-based formulations are added to the vessel, and digestion commences when pancreatic lipase and co-lipase are added. When digestion begins, fatty acids are liberated, which causes a transient drop in pH. A pH electrode coupled to a pH-stat controller and autoburette quantifies the drop in pH, and automatically titrates the liberated fatty acids by adding an equimolar quantity of sodium hydroxide. This maintains the pH at a set point and facilitates indirect quantification of the extent of digestion. Samples are taken over time and centrifuged to separate the digest into an oil phase, an aqueous phase, and a pellet phase, which are then analyzed for drug content [28].

For the Type I-LCF and II-LCF formulations (where an oil phase was present), samples were separated using an ultracentrifuge (4 mL sample volume, 55,000 rpm at 37 °C for 30 min; Optima™ XE-90 Ultracentrifuge, SW60Ti rotor; Beckman Coulter, Palo Alto, CA, USA). For all other formulations, a Thermo Scientific, Heraeus Pico 21 Centrifuge was used (1 mL sample volume, 15 min at 21,000× *g*, 37 °C). The mass of the drug recovered in the aqueous phase and the pellet phase was then quantified by HPLC [35,36]. The in vitro digestion apparatus is shown in Figure 1. The supernatant and pellet samples were diluted with acetonitrile (1:10 *v*/*v*) and then mobile phase (1:10 *v*/*v*), and lumefantrine concentrations in the samples determined using HPLC [35,36,37,38].

**Supersaturation During In Vitro Testing.** The degree of supersaturation of drug concentrations in the aqueous phase during formulation dispersion/digestion was assessed by comparing the measured aqueous phase concentration to equilibrium drug solubility in blank aqueous phase obtained by dispersion and digestion of blank (i.e., non-drug loaded) formulation under identical conditions [39]. Due to the physical state of lumefantrine docusate (i.e., an amorphous semi-solid), the equilibrium solubility assessment was conducted by dissolving 3 mg of IL in 10 µL of ethanol in a microcentrifuge tube and then adding 200 mg of aqueous phase obtained from the blank digestions (n = 3 for each time point for each formulation). The contents were subsequently vortex-mixed for 30 s. Samples were collected at 0.5, 1, 2, 3, and 4 h after addition of blank aqueous phase and then centrifuged (10 min at 14,800 rpm (21,000× *g*) at 37 °C (Thermo Scientific, Heraeus Pico 21 Centrifuge) and assayed by HPLC to determine the drug concentration. Equilibrium solubility was defined when the concentration measured differed by less than 5% across sequential samples. The supersaturation ratio was then calculated as the ratio of the drug concentration measured in the aqueous phase during the dispersion/digestion test to the equilibrium solubility of drug in the aqueous phase at that time point. Supersaturation was assessed at 4 time points during the in vitro dispersion and digestion tests (0 min (end dispersion), and 5, 30, and 60 min (post digestion)) for the Type II-MC, IIIB-MC, IIIB-LC, and IV formulations.

**HPLC Assay Conditions for Lumefantrine.** All HPLC analyses were conducted using a Waters Alliance 2695 Separation Module (Waters Alliance Instruments, Milford, CT, USA). The column was a reverse phase C-18 Phenomenex 3 µm, 100 × 4.6 mm column. The injection volume was 50 µL and UV detection was at 254 nm. The chromatography was run isocratically. The mobile phase consisted of water with 0.1% *w*/*v* formic acid (mobile phase A) and acetonitrile with 0.1% *w*/*v* formic acid (mobile phase B). For quantification of the equilibrium solubility samples, the ratio was fixed at 45:55 *v*/*v* (A/B) while for the in vitro dispersion and digestion samples, the ratio was 50:50 *v*/*v* (A/B). The flow rate was 1 mL/min and the retention times were ~2.5 and 5.3 min respectively. The calibration standards were 50, 200, 1000, 2000, 5000, 10,000, and 20,000 ng/mL.

Validation of the lumefantrine HPLC assay was run over two days. Intra-assay accuracy was determined by replicated analysis (*n* = 5) of three standards at the lowest, middle, and highest concentrations (50, 5000, and 20,000 ng/mL). Inter-assay accuracy was determined on two separate days. The data were expressed as a percentage of the measured concentration over the theoretical concentration. The mean accuracy of the lowest concentration (50 ng/mL) was within ±15% of the theoretical concentration, while the mean accuracy of the middle and highest concentrations (5000 and 20,000 ng/mL) was within ±10% of the theoretical concentration. Intra-assay precision and inter-assay precision were calculated in both runs for each of the three concentrations and expressed as the coefficient of variation. Precision was within ±10% for all three concentrations. Linearity was performed on the standard curves for each analysis and linearity was accepted when the correlation coefficient (*r*^2^) of the regression line was >0.99.

**Oral Bioavailability Studies.** All procedures were approved by the Monash Institute of Pharmaceutical Sciences Animal Ethics Committee (Approval code 13227. Approval date: 10 April 2014). Experiments were conducted in fasted male Sprague-Dawley rats (240–320 g). The day prior to the study, the rats were anaesthetized with isoflurane and the right carotid artery was surgically cannulated with polyethylene tubing to facilitate blood collection (procedure described previously) [40]. Animals were allowed to recover overnight and were fasted up to 12 h prior to and 8 h after dose administration, with water provided ad libitum. The rats were fed 8 h after dosing. The general approach to the doses chosen for the in vivo studies was based on the desire to provide evidence of utility of the IL approach and to provide a comparison between solution and suspension formulations. In general, the potential drug load that could be dissolved using the IL was much higher than that that could be achieved with the free base, and so to compare exposure at a fixed dose (and typically a relatively high dose since this was possible with the IL technology), the IL formulations were solutions whereas the free base formulations were suspensions. However, data were also collected (where possible) at a fixed dose when both free base and IL form were in solution. These studies had to be conducted at a lower dose to allow the free base to be in solution. The compounds used were employed as model low aqueous solubility drugs and therefore the absolute doses chosen were not chosen to have any pharmacological relevance.

The LBFs containing lumefantrine docusate were dispersed (250 mg LBF in 1 mL of water) immediately prior to oral gavage to lightly anaesthetized rats. Animals were then administered a further 0.5 mL of water. The lumefantrine docusate was dissolved in Type II-MCF, Type IIIB-MCF, Type IIIB-LCF, and Type IV formulations to provide a dose of 50, 65, 65, or 85 mg/kg lumefantrine free base, respectively. These doses were equivalent to 80% of equilibrium solubility of lumefantrine docusate in the respective LBFs. Lumefantrine free base was administered as a suspension in the Type IIIB-LCF (i.e., what was expected to be the most effective LBF) at a dose of 85 mg/kg, and dosed as an aqueous suspension at 85 mg/kg. The aqueous suspension comprised 0.5% *w*/*v* sodium carboxymethylcellulose, 0.4% *w*/*v* Tween 80, and 0.9% NaCl in water.

Blood samples were collected via the carotid artery cannula at pre-dose, 0.5, 1, 2, 3, 4, 6, 8, 10, and 24 h after oral administration of the formulations. Blood samples were centrifuged at 6700× *g* for 5 min and plasma was collected and stored at −20 °C until assayed by LC-MS. Statistically significant differences between formulations were determined by one-way analysis of variance (ANOVA) at a test significance level of α ≤ 0.05, followed by a Tukey’s multiple comparisons test.

**Plasma Sample Preparation and Analysis.** Lumefantrine concentrations in rat plasma were assayed via LC-MS using a modification of a previously published assay for lumefantrine [41]. Calibration standards of lumefantrine were prepared by spiking blank rat plasma (50 µL) with lumefantrine standard solutions (10 µL) in 1:1 *v*/*v* water: acetonitrile to give plasma concentrations in the range of 50 to 5000 ng/mL. Plasma samples or calibration standards (50 µL) were spiked with 10 µL of internal standard (halofantrine hydrochloride, 1000 ng/mL in 1:1 *v*/*v* water: acetonitrile) followed by vortex mixing for 10 s. To precipitate plasma proteins, acetonitrile (200 µL) was added and samples vortex mixed for 1 min. The samples were then allowed to stand at room temperature for 30 min. After centrifugation at 10,000× *g* for 10 min at room temperature, 150 µL of supernatant was transferred into vials for analysis.

Lumefantrine plasma samples were analyzed using a Shimadzu LCMS-8050 system (Shimadzu Scientific Instruments, Kyoto, Japan) consisting of a CBM-20A system controller, a DGU-20A5R degassing unit, two Nexera X2 LC-30 AD liquid chromatograph pumps, a Nexera X2 SIL-30AC autosampler, a CTO-20A column oven (held at 40 °C), and a triple quadrupole mass spectrometer with an electrospray ionization (ESI) interface. Data acquisition and processing was performed using LC-MS Solutions software (Shimadzu, Kyoto, Japan). The desolvation line (DL) and heat block were kept at 250 °C and 400 °C, respectively. The nebulizing gas flow and drying gas flow rates were 3.0 L/min and 10.0 L/min, respectively. Mobile phase A was Milli-Q water containing 0.1% *w*/*v* formic acid, and mobile phase B was acetonitrile containing 0.1% *w*/*v* formic acid. The mobile phase flow rate was 0.5 mL/min with the following gradient elution: mobile phase B was first held at 30% for 1.25 min, then linearly increased to 80% over the next 0.75 min. Mobile phase B was then held at 80% for 0.5 min, followed by a linear decrease to 30% over the next 0.5 min after which conditions were held for another 0.5 min. Each sample (1 µL) was injected onto a Phenomenex Kinetex^®^ C18 column (2.6 µm, 100 Å, 50 × 2.1 mm, Sydney, NSW, Australia). The retention times of lumefantrine and the internal standard (halofantrine hydrochloride) were 2.1 and 1.9 min, respectively. Lumefantrine detection was achieved using positive electrospray ionization with multiple-reaction monitoring (MRM) of the 530.2 > 512.2 mass/charge ion peak (*m/z*) at a collision energy of −25.0 V. The internal standard halofantrine hydrochloride was monitored at 500.2 > 142.0 *m/z* at a collision energy of −25.0 V. Sample concentrations were determined by comparison to a calibration curve obtained by fitting the peak area ratio of lumefantrine to internal standard (halofantrine hydrochloride) versus concentration data to a linear equation with a weighting factor inversely proportional to the standard concentration.

Validation of the lumefantrine LC-MS plasma assay was run over two days. Intra-assay accuracy was determined by replicate analysis (*n* = 5) of three standards at the lowest, middle, and highest concentrations (50, 2000, 5000 ng/mL). Inter-assay accuracy was determined on two separate days. The data were expressed as a percentage of the measured concentration over the theoretical concentration. The mean accuracy was within ±10% of the theoretical concentration. Intra-assay precision and inter-assay precision were calculated for both runs for each of the three concentrations and expressed as the coefficient of variation. Precision was within ±10% for all three concentrations. Linearity was performed on the standard curves for each analysis and linearity was accepted when the correlation coefficient (*r*^2^) of the regression line was >0.99.

## 3. Results

### 3.1. Characterization of Lumefantrine Compounds

The measured melting temperatures of the lumefantrine ILs and related compounds are listed in Table 2. Detailed NMR spectroscopy data are listed in the experimental section. The melting point of the lumefantrine ILs appeared to decrease with increasing bulk/complexity of the counterion. Lumefantrine hydrochloride is a yellow powder and shows birefringence under polarized light microscopy, indicating crystallinity. As such, it has the highest melting point (180–200 °C). Lumefantrine free base and lumefantrine dodecyl sulfate are also yellow powders which show some degree of birefringence under the cross-polarized light microscope, but have lower melting points than the hydrochloride salt (133–140 °C and 120–128 °C, respectively). Since the melting point of lumefantrine dodecyl sulfate was >100 °C, it is more appropriately termed a lipophilic salt rather than an ionic liquid [33]. As expected, the complexity of the docusate counterion resulted in isolation of lumefantrine docusate as a clear yellow semi-solid with a much lower melting range of 52–60 °C. Several attempts were made to recrystallize lumefantrine docusate but a crystal form could not be isolated.

### 3.2. Lumefantrine Equilibrium Solubility in Lipid-Based Formulations

**Figure 2:**
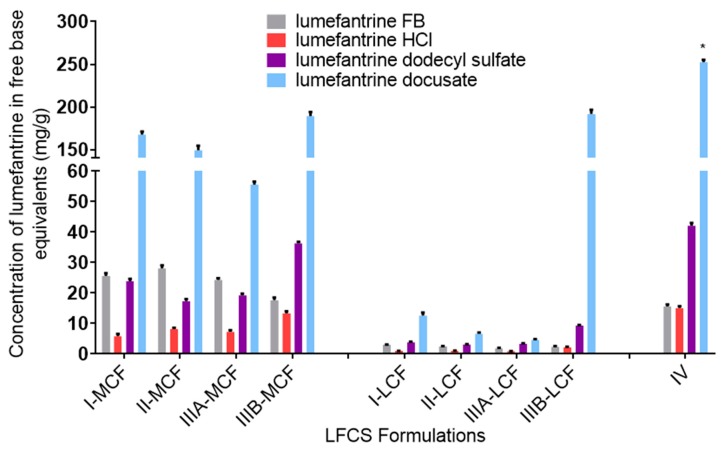
Equilibrium solubility of lumefantrine compounds in lipid-based formulations. Data are expressed in lumefantrine free base equivalents. Data are mean ± SD, *n* = 3. * Lumefantrine docusate was miscible in the Type IV formulation and the data shown reflect the measured concentration after mixing the docusate and formulation in a 1:1 *w*/*w* ratio.

The equilibrium solubility of the lumefantrine compounds was assessed in each of the LBFs described in Table 1. The equilibrium solubility of lumefantrine, lumefantrine hydrochloride, lumefantrine dodecyl sulfate, and lumefantrine docusate in the different LBFs is shown in Figure 2, where the data are presented as the equivalent concentration of free base. In general, lumefantrine solubility was higher in the medium-chain formulations than in the long-chain formulations, and solubility was higher in the more polar formulations such as the Type IIIB and Type IV formulation. This is consistent with previous work, with both non-IL drugs and ILs [32,35]. Isolation of lumefantrine as the dodecyl sulfate IL resulted in increases in solubility in the Type IIIB and Type IV formulations, but did not result in significant advantages in lipid solubility in the others. In contrast, isolation as the docusate IL markedly enhanced solubility in all LBF (2-to-80-fold higher than lumefantrine, depending on formulation) [35].

### 3.3. In Vitro Evaluation of Lumefantrine and Lumefantrine Docusate in LBF

**Figure 3:**
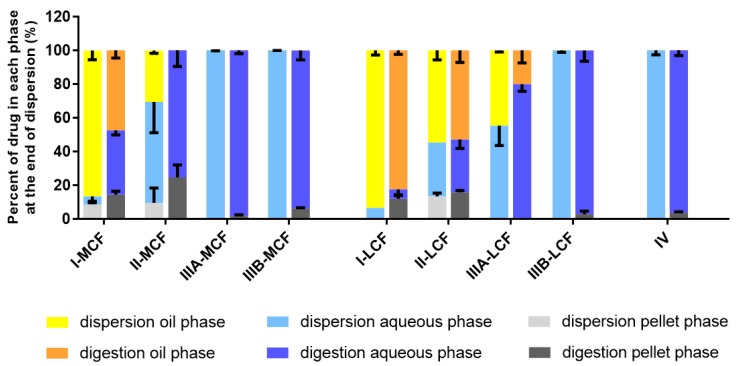
Drug distribution of lumefantrine during in vitro dispersion and digestion tests of LBFs. Data presents % drug in each phase at the end of dispersion phase and at the end of digestion phase. Data are mean ± SD, *n* = 3.

**Figure 4:**
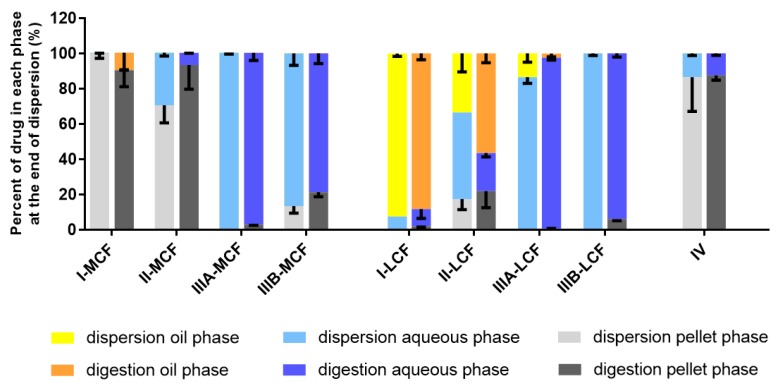
Drug distribution of lumefantrine docusate during in vitro dispersion and digestion tests. Data presents % drug distribution into each phase at the end of dispersion phase and at the end of digestion phase. Data are mean ± SD, *n* = 3.

**Figure 5:**
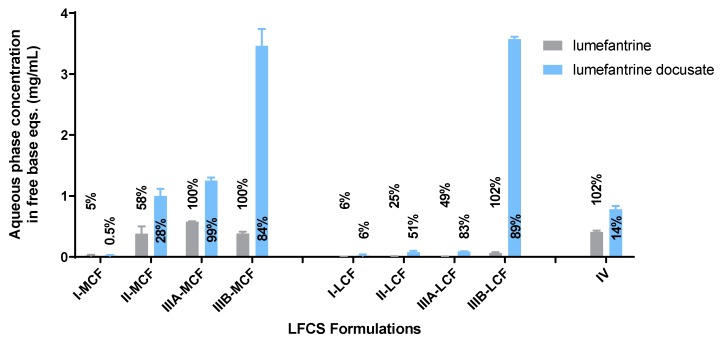
Aqueous phase concentrations of lumefantrine (in free base equivalents) at the end of dispersion phase for LBFs containing lumefantrine or lumefantrine docusate. Percentages are a reflection of the amount of drug present in the aqueous phase compared to total drug loading. Data are mean ± SD, *n* = 3.

**Figure 6:**
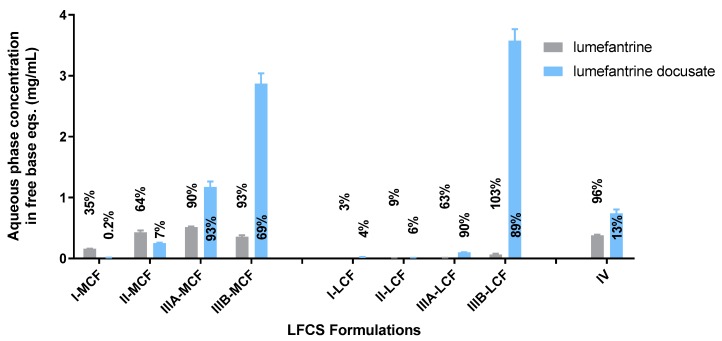
Aqueous phase concentration of lumefantrine (in free base equivalents) at the end of digestion phase for LBFs containing lumefantrine or lumefantrine docusate. Percentages are a reflection of the amount of drug present in the aqueous phase compared to total drug loading. Data are mean ± SD, *n* = 3.

In light of the lower improvement in lipid solubility for the dodecyl sulfate IL, in vitro dispersion and digestion tests were conducted for LBF containing lumefantrine free base and lumefantrine docusate only. The drug-phase distributions for lumefantrine and lumefantrine docusate are shown in Figure 3 and Figure 4, respectively. A comparison of the aqueous phase concentrations attained for LBF containing lumefantrine and lumefantrine docusate (at loading levels of 80% of equilibrium solubility) at the end of the dispersion and digestion phase of experiments is provided in Figure 5 and Figure 6, respectively.

After centrifugation, samples were separated into an oil phase (if present), an aqueous phase, and a pellet phase (all individual in vitro dispersion and digestion data are provided in the Supporting Information). For both lumefantrine and lumefantrine docusate, an oil phase was present more commonly after dispersion and digestion of the long chain lipid-containing formulations (LCF) and was only present for the Type I medium chain formulation (MCF). The Type I formulations contain only lipids and no surfactant and as such disperse poorly resulting in phase separation. Initiation of digestion, however, results in the generation of more amphiphilic digestion products that promote dispersion and recovery in the aqueous phase and reduce drug recovery in the oil phase. Dispersion of the Type II-MCF resulted in the recovery of a large proportion of the drug in the ‘pellet’ phase; however, this likely reflects phase separated (dense) lipid rather than drug precipitation [42]. This suggestion is consistent with a significant reduction in drug recovery in the pellet after digestion is initiated (where digestion results in a change to the nature of the oil phase, an increase in amphiphilicity, and enhancement in dispersion). For both long and medium chain formulations, the Type IIIA and IIIB formulations resulted in good solubilization after both dispersion and digestion and the majority of the drug was present in the aqueous phase. The only significant differences in performance with respect to formulation class were the Type I-MCF, Type II-MCF and Type IV formulations, where significantly more drug precipitation was apparent for the lumefantrine docusate formulation when compared to lumefantrine. This likely reflects the much higher drug loading afforded by the use of the docusate IL.

The absolute aqueous phase concentrations (rather than percent distribution) obtained after dispersion and digestion of the different formulations are provided in Figure 5 and Figure 6, respectively. The concentrations reflect the product of the drug loading in the formulation (which in turn is dictated by the solubility data in Figure 2) and the percent solubilized in the aqueous phase. In all cases, the greater solubility of lumefantrine docusate in the different LBF resulted in higher drug concentrations in the aqueous phase (2-to-55-fold higher) than the equivalent lumefantrine free base formulations. The highest aqueous phase concentrations were attained for the medium and long-chain Type IIIB formulations. This reflects the formulations with the highest drug solubility and good ongoing solubilization under digestion conditions. The Type IIIA formulations resulted in good solubilization post digestion, but were limited by drug solubility in the formulation, and the Type IV formulation showed good solubility in the formulation, but more extensive precipitation post digestion.

### 3.4. Supersaturation Ratio Calculation for Lumefantrine

**Figure 7:**
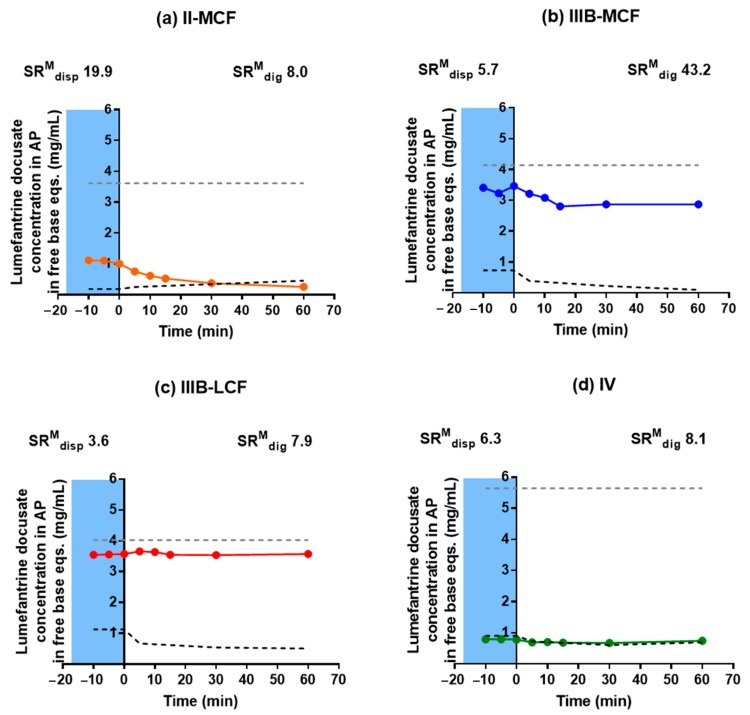
Drug concentrations during in vitro tests (colored circles) of (**a**) Type II-MCF (orange), (**b**) Type IIIB-MCF (blue), (**c**) Type IIIB-LCF (red), and (**d**) Type IV formulations (green). The upper grey line represents the *AP_max_*, and the lower dotted black line represents the equilibrium solubility of lumefantrine docusate in the aqueous colloidal phase of blank LBF. The blue shaded section denotes the dispersion phase. Data are mean ± SD, *n* = 3. The *SR^M^* at time 0 min (*SR^M^_dig_*) and 60 min (*SR^M^_dig_*) min are displayed for each formulation.

The supersaturation ratio (SR) provides an indication of thermodynamic activity and the likelihood of either drug precipitation, or enhanced absorption from an LBF, where the higher the number, the more supersaturated and therefore the more likely to precipitate during in vitro digestion. The maximum supersaturation ratio (*SR^M^*) provides a measure of the maximum supersaturation pressure that would be generated by digestion of an LBF, assuming no precipitation and is calculated from the following:

(1) $$SR^{M}=\frac{AP_{max}}{AP_{digest}}$$

where *AP_max_* is the maximum possible aqueous phase concentration of drug during the in vitro dispersion and digestion test (i.e., in the absence of any precipitation), and *AP_digest_* is the equilibrium solubility of the drug in blank aqueous phase (i.e., where the aqueous phase was obtained from a digestion of blank LBF). Williams et al. have previously proposed that sustainable supersaturation most commonly occurs when *SR^M^* < 3, and that exceeding this threshold increases the likelihood of drug precipitation during in vitro dispersion and digestion tests [43]. The concentrations of lumefantrine docusate during in vitro tests for the four formulations are shown in Figure 7. Only the Type II-MC, IIIB-MC, IIIB-LC, and IV formulations were evaluated as these were the four formulations chosen to proceed to the in vivo bioavailability studies. The figures also depict *AP_max_*, as well as the drug solubility in the aqueous colloidal phase of the blank LBF (*AP_digest_*). In all cases, *SR^M^* was higher than 3, suggesting the potential for precipitation. This was most apparent at early time points for the Type II-MCF formulation where *SR^M^* was ~20 and at later time points post digestion for the Type IIIB-MCF where *SR^M^* was >40. However, unlike previous studies, here the *SR^M^* did not appear to correlate well with drug precipitation (i.e., the presence of drug in the pellet phase). For example, while the *SR^M^* during dispersion was lowest for the Type IIIB-LCF and Type IIIB-MCF, and these two formulations appeared to most robustly resist precipitation, closer analysis of the data shows that for the Type IIIB-LCF, the *SR^M^* at the end of digestion was similar to that of the Type II-MCF and Type IV (where precipitation was significant), but the Type IIIB-LCF was able to maintain drug solubilization throughout the test (89% drug solubilized). Similarly, the *SR^M^* for the Type IIIB-MCF at the end of digestion was the highest, but majority of lumefantrine docusate remained solubilized at 60 min (69% solubilized).

**Figure 8:**
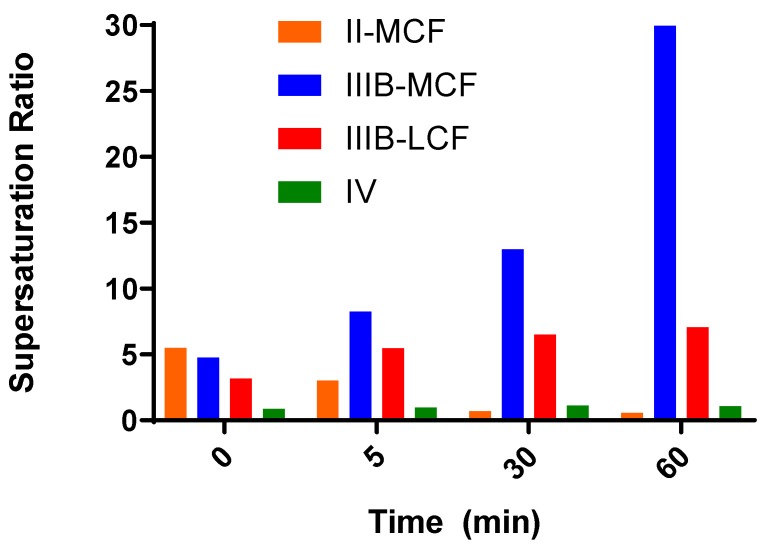
The supersaturation ratios for Type II-MC, Type IIIB-MC, Type IIIB-LC, and Type IV formulations, calculated from the aqueous phase concentrations of the drug during in vitro dispersion and digestion tests at time points of 0, 5, 30, and 60 min divided by the equilibrium solubility of lumefantrine docusate in aqueous colloidal phase of blank LBF at the same times (using data from Figure 7).

Supersaturation data at 0, 5, 30, and 60 min are shown in Figure 8. The supersaturation ratios in Figure 8 were calculated via the ratio of the concentration of drug measured in the aqueous phase during the in vitro tests compared to the drug solubility in the colloidal aqueous phase at the same time point. This gives an indication of the degree of supersaturation of the solubilized drug concentrations throughout the in vitro test, rather than the maximum degree of supersaturation. The Type II-MCF had a reasonably high SR during dispersion (5.5) and consistent with an SR > 3, the majority of the drug was present in the pellet phase which was likely a mix of phase separation and precipitation. On initiation of digestion, precipitation continued, and the solubilized concentration dropped towards the equilibrium solubility (Figure 7). As such, supersaturation was low for the rest of the test (Figure 8). Similarly, the Type IV formulation dispersion led to an *SR^M^* of 6.3 and significant precipitation on dispersion and digestion. In this case, precipitation to the equilibrium solubility occurred almost immediately and no supersaturation was evident throughout the in vitro test. In contrast for both Type III formulations, solubilization and supersaturation were maintained to varying degrees throughout the in vitro test. For the IIIB LC formulation, *SR^M^* on dispersion was quite low (3.6), and close to the previously described limit for precipitation. Consistent with these previous studies, drug precipitation from this formulation was low and moderate levels of supersaturation were maintained throughout formulation dispersion and digestion. The degree of supersaturation gradually increased throughout the test. For the Type IIIB MC formulation, *SR^M^* on dispersion was slightly higher (5.7) and some precipitation was evident. However, precipitation was still quite low and drug solubilization and supersaturation were maintained. Indeed, supersaturation increased significantly throughout the digestion period due to a fall in equilibrium solubilization capacity. Thus, solubilization and supersaturation were highest for the Type IIIB formulations and amongst these, supersaturation was seemingly higher for the Type IIIB MC formulation.

### 3.5. In Vivo Evaluation of Lumefantrine Docusate

**Figure 9:**
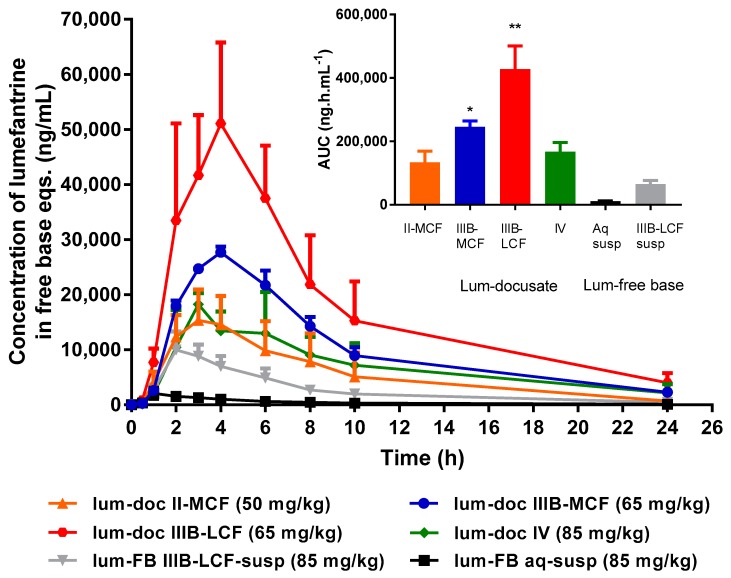
Lumefantrine in vivo bioavailability study. Lumefantrine plasma concentration versus time after oral administration of lumefantrine docusate in Type II-MCF, IIIB-MCF, IIIB-LCF, and IV LBF, as well as lumefantrine free base as an aqueous suspension, and a lipid suspension (in the Type IIIB-LCF). Lumefantrine docusate in Type II-MCF was dosed at 50 mg/kg. Lumefantrine docusate in Type IIIB-MCF and IIIB-LCF were dosed at 65 mg/kg. Lumefantrine docusate in Type IV formulation and the free base suspensions were dosed at 85 mg/kg. Data have been dose normalized to the nominal (i.e., 50–85 mg/kg) dose. Data represented as mean (*n* = 4) ± SEM. Insert: The total exposure over 24 h, measured as area under the plasma concentration versus time curve from 0 to 24 h. Data are mean ± SD (*n* = 4). * Formulation was statistically significant (*p* < 0.05) when compared to both suspensions. ** Formulation was statistically significant (*p* < 0.05) from all other formulations.

After screening the formulations through in vitro dispersion and digestion testing, four lumefantrine docusate formulations were chosen to progress to an in vivo bioavailability study (Type II-MCF, IIIB-MCF, IIIB-LCF, and IV), and the data are shown in Figure 9. The dose for each IL formulation was set at ~80% of the equilibrium solubility value described in Figure 2. The doses of lumefantrine docusate in Type II-MCF, IIIB-MCF, IIIB-LCF, and IV formulations (in free base equivalents) were 50 mg/kg, 65 mg/kg, 65 mg/kg, and 85 mg/kg, respectively. Due to the low lipid solubility of lumefantrine free base, the free base was dosed as a suspension in aqueous and lipid (Type IIIB LCF) formulations at an equivalent dose to the highest IL-LBF (85 mg/kg, attained in the Type IV formulation). The suspension formulations significantly underperformed the equivalent lipid solution formulations made possible by the presence of lumefantrine docusate. Data dose normalized to a fixed nominal dose of 50 mg/kg are shown in the supplementary information, however the major conclusions are unchanged since the two best performing formulations (Type IIIB) were administered at the same dose. The non-normalized data also serve to show the maximal benefit of the IL formulations since this is derived from the ability to both increase dose and harness the advantages of a LBF.

## 4. Discussion

**Physicochemical Properties of Lumefantrine Compounds.** As expected, the melting point of lumefantrine hydrochloride (180–200 °C) was the highest of the lumefantrine salts, followed by lumefantrine free base (133–140 °C), lumefantrine dodecyl sulfate (120–128 °C) and lumefantrine docusate (52–60 °C). The hydrochloride salt, free base, and dodecyl sulfate salt showed birefringence under cross-polarized light, indicating crystallinity, while the docusate-IL did not display birefringence, indicating that the material was amorphous. This property is reflected in the differences in melting point, with the crystalline materials having higher melting points. Higher melting compounds typically have stronger electrostatic forces holding the ions together. In these studies, the molecular bulk of the dodecyl sulfate and docusate counterions was expected to decrease packing efficiency, decrease the strength of intermolecular interactions and therefore decrease the melting point, and this appeared to occur [18]. The greater reduction with the docusate counterion is consistent with a higher molecular weight and enhanced steric bulk of the docusate counterion resulting in a greater disruption of the packing of the crystal lattice than the dodecyl sulfate or the hydrochloride. The difference in melting point between lumefantrine dodecyl sulfate and lumefantrine docusate is similar to the difference between the melting points of the sodium salts of the counterions of ~60 °C (sodium dodecyl sulfate melting point is 205.5 °C, [44,45] sodium docusate melting point is 153–157 °C) [45,46].

**Effect of Formulation Type on LBF Solubility of Lumefantrine Compounds.** The equilibrium solubility of lumefantrine free base was higher in the more lipophilic formulations (Type I and II formulations), while the HCl salt, dodecyl sulfate salt, and docusate IL all had higher solubilities in the more hydrophilic formulations (Type IIIB and IV formulations). The docusate IL was miscible in the Type IV formulation (i.e., soluble at >1:1 *w*/*w* proportions, the data in Figure 2 reflecting the measured concentration after mixing at a 1:1 ratio). This trend is consistent with previous work, suggesting greater affinity of the drug for the surfactant and co-solvent rich Type III and IV formulations rather than the Type I or Type II lipid rich formulations [35,47]. Thus, even though pairing with highly lipophilic counterions to form an IL might be expected to increase the affinity of lumefantrine for the more lipid rich formulations, the trends in relative solubility across formulation type were similar to that of lumefantrine HCl, and the solubility was higher in the more polar formulations. Surprisingly, lumefantrine dodecyl sulfate only resulted in a solubility advantage compared to lumefantrine free base in the Type IIIB and Type IV formulations, and these increases were only moderate. This may suggest that the dodecyl chain is unable to disrupt packing in the crystal lattice sufficiently to impact solubility significantly. This is consistent with the fact that the reduction in melting point for the dodecyl sulfate was not as pronounced as for the docusate-IL (where solubility was much higher in all formulations). As has been described previously for other drugs, for all lumefantrine salts, drug solubility was higher in the medium-chain formulations when compared to long-chain formulations [48]. This has previously been suggested to reflect the fact that for a fixed mass of lipid, medium-chain lipids contain a greater number of moles of lipid than the long-chain equivalent and drug solubility in lipids appears to be related to the number of ester bonds present in the lipids [48].

**Behavior of LBF of Lumefantrine Free Base and Lumefantrine Docusate in vitro.** The relatively moderate changes to solubility apparent with the dodecyl sulfate salt dictated that further analysis was conducted only with the docusate IL in comparison to lumefantrine free base. Interestingly, the relative solubilization/partition behavior of most of the LBF of lumefantrine and lumefantrine docusate were quite similar after dispersion and digestion, in spite of the much higher drug loading of the IL-containing formulations. For the Type I-MCF, II-MCF and IV formulations, however, differences were evident and a greater proportion of drug was present in the pellet phase for the (higher loaded) lumefantrine docusate formulations. More detailed discussion of the in vitro solubilization trends is provided below.

Type I LBFs are composed of lipids alone and were not fully digested at the end of the in vitro experiment. The lack of surfactant in the formulation also reduced dispersion, resulting in the majority of lumefantrine free base residing in the oil phase of the MCF. In contrast, for the docusate IL, the majority of the drug was present in the pellet phase after digestion of the Type I MCF. This is likely due to phase separation of amorphous lumefantrine IL, rather than precipitation and may reflect the higher loading of drug. Additionally, digestion of medium-chain lipids may yield hydrophobic digestion products which retain the solubilization capacity of the digested formulation, but are denser than the aqueous layer and therefore centrifuge to the bottom of the tube [40]. For both the free base and the docusate IL, the majority of the drug was also present in the oil phase after digestion of the Type I-LCF. In this case, however, this was due to incomplete dispersion and digestion of the long-chain lipids.

The Type II formulations contain a lipophilic surfactant, Tween 85^®^, which is denser than water. This density difference resulted in a phase separation of the formulation during the dispersion phase for the Type II formulations and recovery of a large proportion of drug in a dense oily pellet phase, particularly for the IL in Type II-MCF. The magnified effect with the IL containing formulation again likely reflects the higher drug loading. Upon digestion, drug distribution across the different phases did not change significantly relative to the dispersion phase, although there was an increased proportion of drug in the pellet phase. As such, drug concentrations in the aqueous phase were low. This increased proportion of drug in the pellet phase is likely a combination of phase separation and precipitation, due to both the denser surfactant and the increased drug loading.

The Type IIIA and IIIB formulations resulted in markedly improved drug solubilization during formulation dispersion and digestion and more than 80% of drug remained solubilized, regardless of the use of lumefantrine or lumefantrine docusate or medium or long-chain lipids. As such, the aqueous phase concentrations obtained were driven by drug solubility in the formulation—which was highest for the Type IIIB formulations, and significantly higher for the IL based formulations relative to the free base.

Type IV formulations are composed entirely of co-solvent and surfactant and resulted in the highest drug solubility in the formulation. The majority of lumefantrine free base remained solubilized at the end of the digestion phase for the Type IV formulation. However, for lumefantrine docusate, there was significantly increased drug precipitation, presumably reflecting the much higher drug loading in the formulation. Type IV formulations containing lumefantrine docusate resulted in substantial precipitation upon dispersion, likely as a result of dilution of co-solvent and surfactant and loss of solvent capacity on dilution. Digestion of the surfactant, Kolliphor^®^ RH 40, may also have caused a further decrease in solubilization capacity. Nonetheless, the much higher drug loading of the docusate-IL in the Type IV LBF resulted in a net effect of much higher aqueous phase drug concentrations when compared to the free base, despite the increased precipitation.

*SR^M^* is an indicator of the propensity of a formulation to precipitate during in vitro dispersion and digestion, where the higher the *SR^M^*, the more likely a formulation is to precipitate. In contrast where the *SR^M^* is below 3, previous results suggest that the formulation is more likely to exhibit sustained supersaturation [41]. This in turn is more likely to generate conditions where absorption is favored in vivo; even relatively brief periods of high supersaturation have been shown to very effectively support absorption for highly permeable drugs [47,49].

The Type II-MCF had the highest *SR^M^* during the dispersion phase, which then decreased upon digestion. The high apparent supersaturation during dispersion is consistent with the majority of the drug being recovered in the pellet phase, which was likely a mix of phase separation and precipitation. The high density of Tween 85^®^ leads to phase separation of surfactant and therefore decreased solubilization capacity. Digestion did not improve the solubilization capacity as the concentration of solubilized drug dropped to the equilibrium solubility of the drug, and therefore the apparent supersaturation was low for the remainder of the in vitro test.

The medium and long chain Type IIIB formulations had a lower *SR^M^* during dispersion, but this increased upon digestion. Both formulations were able to maintain drug solubilization throughout the in vitro test. The degree of apparent supersaturation increased as the in vitro test progressed, with the degree of supersaturation of the Type IIIB-MCF increasing at a greater rate than the LCF counterpart. As medium-chain lipids are more readily digested than the equivalent long-chain lipids [50], the solubilization capacity for medium-chain formulations is lost more quickly than for long-chain formulations, and therefore the degree of supersaturation is greater. The Type IIIB-LCF was able to retain a greater proportion of drug in the solubilized state, consistent with the lower degree of supersaturation compared to the Type IIIB-MCF. In both cases, solubilization was the highest for the Type IIIB formulations. The Type IV formulation had a similar solubilization profile to the Type II-MCF. The *SR^M^* upon dispersion was 6.3, which then increased to 8.1 at the end of digestion. Unlike the Type IIIB formulations, however, the majority of the drug precipitated out during dispersion of the Type IV formulation and concentrations remained at equilibrium solubility throughout the in vitro test. As such, no supersaturation was observed.

Although supersaturation is a driver of precipitation in vitro, increases in supersaturation in vivo result in increases in thermodynamic activity and may therefore result in increases in absorption. As such, whether supersaturation results in an increase or a decrease in absorption is typically a trade-off between the drivers of precipitation and absorption. The in vitro data suggest that the Type III formulations, where both solubilization and supersaturation were maintained, were most likely to promote absorption in vivo. This suggestion was subsequently probed via the conduct of an in vivo bioavailability study.

**Effect of Formulation Type on in vivo Bioavailability of Lumefantrine Docusate.** Oral administration of all four lumefantrine docusate IL-containing solution LBFs resulted in higher systemic exposure than that obtained after oral administration of aqueous or lipid suspensions of lumefantrine free base (Figure 9). Statistical analysis by one-way ANOVA suggest significantly higher exposure after administration of the docusate IL containing Type IIIB-MCF compared to both free base suspensions, while the Type IIIB-LCF exhibited significantly higher exposure than all other formulations tested. Administration of the lumefantrine docusate Type IIIB-LCF resulted in the highest plasma concentrations, followed by the Type IIIB-MCF. The Type IV and II-MCF formulations had similar and intermediate exposure profiles, and these were both higher than the lipid suspension and aqueous suspension of the free base. Figure 9 shows these trends and illustrates that the IL containing formulations that are able to dissolve higher quantities of drug and therefore facilitate the administration of higher doses also result in higher exposure. The data also show that for lumefantrine, lipid suspension formulations were unable to provide the same benefits to exposure apparent with the IL containing lipid solution formulations. The profiles were also normalized to the same dose, to remove the effects of differing dose and to instead look at the intrinsic absorption promoting ability of the formulations at a fixed dose. Surprisingly, however, this did not markedly change the conclusions, except to further relegate the Type IV formulation where dose was high, but precipitation was also very high. Thus, although the Type IV formulation allows for the highest drug loading, the high dose is not enough to overcome the precipitation that occurs upon administration (and indeed may stimulate it). The exposure obtained after administration of the Type II-MCF was also relatively poor, reflecting both low drug loading and low absorption enhancement. In contrast, and broadly consistent with the in vitro data, both Type IIIB formulations resulted in significantly higher lumefantrine exposure after oral administration. The use of the IL therefore allowed for both higher doses and enhanced exposure (up to 50-fold) when compared to the free base.

Correlating *in vitro-in vivo* performance is complex as there are many factors which affect performance. For example, the lack of an absorption sink in vitro usually results in overestimation of precipitation [38,50]. Choice of in vivo model (i.e., rat, pig, dog, etc.) provides an additional layer of complexity as each model has physiological differences that make prediction of the eventual translation to humans difficult [51]. Nevertheless, there have been examples of lipid formulations (including those that contain IL) that do show a strong *in vitro/in vivo* correlation. For example, Sahbaz et al. have reported that combination of cinnarizine decyl sulfate with a SEDDS formulation resulted in ~2-fold higher exposure in rats when compared to an equivalent dose of cinnarizine free base as a suspension in the same SEDDS formulation. Similarly, itraconazole docusate in a SEDDS formulation resulted in ~20-fold higher exposure in rats than an itraconazole suspension of equal dose, and exhibited greater exposure than the commercial formulation Sporanox^®^. For both cinnarizine decyl sulfate and itraconazole docusate, the enhanced performance of the IL in vivo was consistent with improved in vitro solubilization on formulation dispersion and digestion when compared to the corresponding free base suspensions [32].

Williams et al. have also reported that lipophilic salt forms of small molecule kinase inhibitors exhibit higher solubility in lipidic excipients when compared to the free base or commercial salt form. In this example, increased solubility resulted in increased drug loading of the small molecule kinase inhibitors in LBF. Isolation of erlotinib and cabozantinib as lipophilic salts (erlotinib docusate and cabozantinib docusate) also led to increased aqueous phase concentrations in vitro at gastric pH when compared to an aqueous suspension of the corresponding hydrochloride salt. Under intestinal conditions where pH increased and digestion was stimulated, solubilized drug concentration dropped significantly, however the IL formulations were still able to provide solubilization advantage when compared to the free base. *In vivo,* LBF containing erlotinib docusate resulted in lower variability and better dose linearity when compared to an aqueous suspension of the (commercial) hydrochloride salt. Cabozantinib docusate also resulted in improvements in exposure when formulated in an LBF and bioavailability was enhanced 1.5- and 1.8-fold after administration of a medium-chain SEDDS and long-chain SEDDS compared to an aqueous suspension of cabozantinib free base, respectively [52].

**Figure 10:**
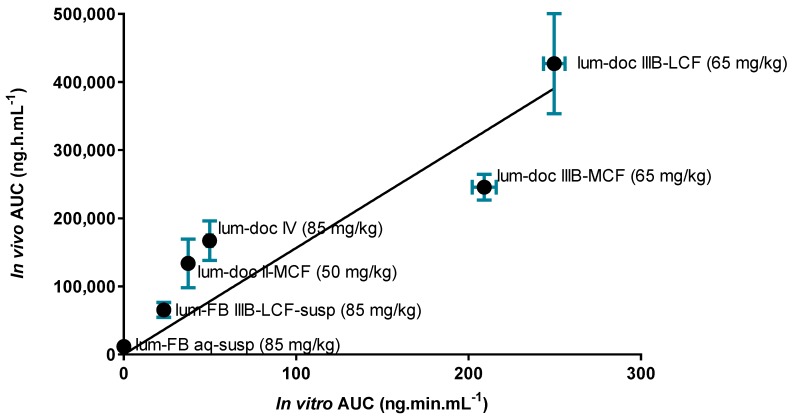
In vitro/in vivo correlation plot displaying the AUC for the aqueous solubilization during in vitro dispersion and digestion experiments, and the AUC for in vivo exposure. Data are expressed as mean ± SD for in vitro AUC (*n* = 3) and mean ± SEM for in vivo AUC (*n* = 4).

To more carefully analyze the relationship between in vitro and in vivo endpoints, Figure 10 shows the correlation between the degree of drug solubilization during in vitro dispersion and digestion (expressed as the AUC of the drug concentration in the digest aqueous phase over time) and in vivo exposure (expressed as the AUC of the plasma drug concentration versus time profile after oral administration). The strength of the correlation suggests that increases in drug solubilization translate to increases in drug absorption and exposure. The drug dose/load in the formulations in both the in vitro and in vivo tests varied across formulations, but was the same in each test (i.e., in vitro and in vivo) for each formulation. According to the Pearson coefficient (2-tailed test), the strength of the association between the in vitro performance and in vivo performance is high (r^2^ = 0.8695), and the correlation is significant (*p* < 0.01).

For poorly water-soluble drugs, increases in drug solubilization typically promote drug absorption as shown in Figure 10. In addition to drug solubilization, previous studies have also shown that differences in the degree of drug supersaturation (rather than solubilization) may be important drivers of absorption [45,47]. In the current studies, however, this appears not to be the case as there is no correlation between supersaturation and drug exposure (Supplementary Figure S20). The data therefore suggest that for lumefantrine, solubilization behavior appears to be a better indicator of in vivo performance (Figure 10) than supersaturation. This trend contrasts with recent studies of similar lipid formulations of fenofibrate, where it was reported that supersaturation was a more significant driver of in vivo exposure [47,49]. However, Crum et al. found that for higher doses of fenofibrate, where longer absorption periods were required, ongoing solubilization was the more significant driver of absorption [47]. As the drug loading for lumefantrine docusate is relatively high, longer absorption times may be required and therefore ongoing solubilization may drive absorption more effectively than supersaturation. The difference in the driving force for absorption may also reflect differences in intrinsic permeability, especially at lower dose. Thus, for drugs which are absorbed quickly, and where permeability is high such as fenofibrate, relatively brief periods of supersaturation may be very effective drivers of in vivo absorption. In contrast, where absorption is slower, permeability is lower and dose is higher, for example with lumefantrine, ongoing solubilization may be more important.

## 5. Conclusions

The use of API-ILs in conjunction with lipid-based formulations has been examined as a means to enhance the oral exposure of lumefantrine. The data suggest significant benefits in solubility in lipid-based formulations and oral exposure are possible using this approach. Isolation as lumefantrine docusate resulted in consistent increases in lipid solubility when compared to lumefantrine free base across a range of lipid-based formulations. The benefits in solubility were subsequently shown to persist and provide for performance advantages during in vitro dispersion and digestion testing, and ultimately, exposure in vivo. The data suggest that using a large, bulky, lipophilic counterion (such as docusate) can both improve the lipid solubility of the parent compound and enhance drug solubilization during formulation processing under simulated GI conditions. For the first time for LBF of ILs, a range of different formulations were explored and the Type IIIB LBFs resulted in the most effective solubilization and supersaturation in vitro and almost completely resisted precipitation. In vivo, these two formulations also significantly out-performed Type II and Type IV formulations of lumefantrine. Within the Type IIIB formulations, the LCF based formulation appeared to support more effective absorption of lumefantrine when compared to the Type IIIB MCF. This was consistent with slightly better drug solubilization for the Type IIIB LCF formulation, but was in contrast to the lower levels of supersaturation when compared to the Type IIIB MC formulation. The outperformance of the LC lipid containing formulation may also reflect improved support for lymphatic transport since the close structural analogue, halofantrine, [53,54] has been shown previously to be highly lymphatically transported, and LC lipids more effectively support lymph transport than medium chain lipids [55]. However, increases in lymphatic transport are thought to increase bioavailability primarily via changes in first pass metabolism and the potential importance of high first pass metabolism relative to low water solubility in driving the low oral bioavailability of lumefantrine is not known. Nonetheless, in all cases, the use of the docusate IL was able to significantly enhance drug loading in lipid formulations, and promote solubilization. In the case of the Type IIIB LC formulation, this solubilization advantage resulted in very large increases in exposure after oral administration when compared to aqueous (~50 fold) or lipid (~10 fold) suspensions of the free base. The data lend further support to the potential utility of ionic liquid drug forms as a means to enhance drug exposure [1,33,56].

## Figures and Tables

**Table 1:** Composition of the lipid-based formulations used for equilibrium solubility and in vitro dispersion and digestion studies. All formulations contained ~1% butylated hydroxytoluene as an anti-oxidant.

Formulation Type	Component	Composition
		(% *w*/*w*)
I-MC	Captex^®^ 355 EP/NF	50.0
	(Glyceryl tricaprylate/caprate)	
	Capmul^®^ MCM EP	50.0
	(Glyceryl monocaprylocaprate)	
II-MC	Captex^®^ 355 EP/NF	32.5
	(Glyceryl tricaprylate/caprate)	
	Capmul^®^ MCM-EP	32.5
	(Glyceryl monocaprylocaprate)	
	Tween™ 85	35.0
	(Polyoxyethylene sorbitan trioleate)	
IIIA-MC	Captex^®^ 355 EP/NF	32.5
	(Glyceryl tricaprylate/caprate)	
	Capmul^®^ MCM EP	32.5
	(Glyceryl monocaprylocaprate)	
	Kolliphor^®^ RH 40	35.0
	(Polyoxyl 35 castor oil)	
IIIB-MC	Capmul^®^ MCM-EP	25.0
	(Glyceryl monocaprylocaprate)	
	Kolliphor^®^ RH 40	50.0
	(Polyoxyl 35 castor oil)	
	ethanol	25.0
I-LC	Maisine™ 35-1	50.0
	(Glyceryl monolinoleate)	
	soybean oil	50.0
II-LC	Maisine™ 35-1	32.5
	(Glyceryl monolinoleate)	
	soybean oil	32.5
	Tween™ 85	35.0
	(Polyoxyethylene sorbitan trioleate)	
IIIA-LC	Maisine™ 35-1	32.5
	(Glyceryl monolinoleate)	
	soybean oil	32.5
	Kolliphor^®^ RH 40	35.0
	(Poloxyl 40 hydrogenated castor oil)	
IIIB-LC	Maisine™ 35-1	25.0
	(Glyceryl monolinoleate)	
	Kolliphor^®^ RH 40	50.0
	(Poloxyl 40 hydrogenated castor oil)	
	ethanol	25.0
IV	Kolliphor^®^ RH 40	50.0
	(Poloxyl 40 hydrogenated castor oil)	
	ethanol	50.0

**Table 2:** The melting point range and appearance under cross-polarized light of lumefantrine free base, the traditional salt lumefantrine hydrochloride and the lipophilic salts lumefantrine dodecyl sulfate and lumefantrine docusate. The lower melting range (<100 °C) of lumefantrine docusate further classifies this material as an ionic liquid.

Compound	Cross-Polarized Light Microscope Image	Melting Point Range (°C)
lumefantrine free base	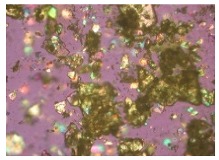	133–140
lumefantrine hydrochloride	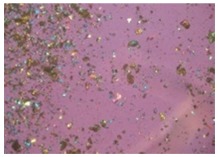	180–200
lumefantrine dodecyl sulfate	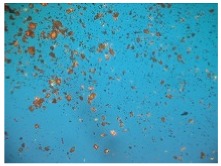	120–128
lumefantrine docusate	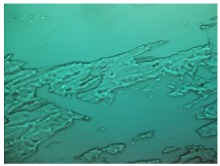	52–60

## Supplementary Materials

The following are available online at https://www.mdpi.com/1999-4923/12/1/17/s1. ^1^H, ^13^C NMR and HR-MS Spectra/Data. Figure S1: In vitro dispersion and digestion of lumefantrine free base in Type I-MCF. The concentration of lumefantrine free base in the aqueous phase of the medium-chain digest as a function of time (left), and the proportion of lumefantrine free base in the pellet, aqueous, and oil phases as a function of time (right). Data are *n* = 3, mean ± SD. * Figure S2: In vitro dispersion and digestion of lumefantrine free base in Type I-LCF. Figure S3: In vitro dispersion and digestion of lumefantrine free base in Type II-MCF. Figure S4: In vitro dispersion and digestion of lumefantrine free base in Type II-LCF. Figure S5: In vitro dispersion and digestion of lumefantrine free base in Type IIIA-MCF. Figure S6: In vitro dispersion and digestion of lumefantrine free base in Type IIIA-LCF. Figure S7: In vitro dispersion and digestion of lumefantrine free base in Type IIIB-MCF. Figure S8: In vitro dispersion and digestion of lumefantrine free base in Type IIIB-LCF. Figure S9: In vitro dispersion and digestion of lumefantrine free base in Type IV. Figure S10: In vitro dispersion and digestion of lumefantrine docusate in Type I-MCF. Figure S11: In vitro dispersion and digestion of lumefantrine docusate in Type I-LCF. Figure S12: In vitro dispersion and digestion of lumefantrine docusate in Type II-MCF. Figure S13: In vitro dispersion and digestion of lumefantrine docusate in Type II-LCF. Figure S14: In vitro dispersion and digestion of lumefantrine docusate in Type IIIA-MCF. Figure S15: In vitro dispersion and digestion of lumefantrine docusate in Type IIIA-LCF. Figure S16: In vitro dispersion and digestion of lumefantrine docusate in Type IIIB-MCF. Figure S17: In vitro dispersion and digestion of lumefantrine docusate in Type IIIB-LCF. Figure S18: In vitro dispersion and digestion of lumefantrine docusate in Type IV. Figure S19: Dose normalized lumefantrine plasma concentration versus time data after oral administration of lumefantrine docusate in Type II-MCF, IIIB-MCF, IIIB-LCF, and IV LBF, as well as lumefantrine free base as an aqueous suspension, and a lipid suspension (in the Type IIIB-LCF). All data have been dose normalized to 50 mg/kg (reflecting the lowest dose administered (Type II-MCF)). Data represented as mean (*n* = 4) ± SEM. Insert: Total lumefantrine exposure over 24 h. Data represented as mean (*n* = 4) ± SEM. * Exposure was statistically higher (*p* < 0.05) than both suspension formulations. ** Exposure was statistically higher than all other formulations. Figure S20: Apparent supersaturation ratio/in vivo correlation plot, displaying the AUC of the apparent supersaturation ratio across the in vitro experiment, and the AUC for in vivo exposure. Data are expressed as mean ± SEM for in vivo AUC (*n* = 4). * The details for Figures S2–S18 are the same, but the extended titles have been left out for the sake of brevity.
