# Supplementary material for: Ionic Liquid Forms of the Antimalarial Lumefantrine in Combination with LFCS Type IIIB Lipid-Based Formulations Preferentially Increase Lipid Solubility, In Vitro Solubilization Behavior and In Vivo Exposure
Source: Pharmaceutics. 2019 Dec 22;12(1):17. doi: 10.3390/pharmaceutics12010017 (PMC7023222; doi:10.3390/pharmaceutics12010017)
Supplement: Supplementary file 1 [file pharmaceutics-12-00017-s001.pdf]

pharmaceutics

Supplementary Materials: Ionic Liquid Forms of the Antimalarial Lumefantrine in Combination with LFCS Type IIIB Lipid-Based Formulations Preferentially Increase Lipid Solubility, In Vitro Solubilization Behavior and In Vivo Exposure

Erin Tay, Tri-Hung Nguyen, Leigh Ford, Hywel D. Williams, Hassan Benameur, Peter J. Scammells and Christopher J. H. Porter

**Supporting Information**		**Page**
**Compound Characterization**		
	^{1}H and ^{13}C NMR data, and HRMS data	2
***In vitro* Data**		
	Drug Distribution and Aqueous Phase Concentrations for Lumefantrine Free Base in Type I-IV Formulations	3
	Drug Distribution and Aqueous Phase Concentrations for Lumefantrine Docusate in Type I-IV Formulations	8
***In vivo* Data**		
	Dose Normalised (all doses 50 mg/kg) Pharmacokinetic Data	13
	In vivo AUC vs. Supersaturation Ratio Correlation Plot	14

^{1}H and ^{13}C NMR data, and HRMS data

Lumefantrine hydrochloride: [$C_{25}H_{30}I_2NO^+$][[Cl^-] ^{1}H NMR (DMSO-*d*$_6$) δ 10.17 (s, 1H), 8.20 (dd, J = 14.0, 3.9 Hz, 3H), 7.67 – 7.57 (m, 5H), 7.48 (dd, J = 8.4, 2.0 Hz, 1H), 7.37 (d, J = 2.0 Hz, 1H), 6.63 (d, J = 4.3 Hz, 1H), 5.95 – 5.85 (m, 1H), 3.44 – 3.37 (m, 1H), 3.29 – 3.27 (m, 1H), 3.27 – 3.10 (m, 3H), 1.77 – 1.58 (m, 4H), 1.41 – 1.26 (m, 4H), 0.91 (t, J = 7.2 Hz, 3H), 0.91 (t, J = 6.8 Hz, 3H). ^{13}C NMR (DMSO-*d*$_6$) δ 141.4, 139.1, 138.2, 135.0, 134.9, 134.4, 133.6, 133.5, 132.5, 131.6, 130.9, 130.5, 129.0, 128.4, 126.9, 125.8, 122.6, 121.1, 64.8, 56.9, 53.3, 52.7, 24.7, 24.5, 19.5, 19.4, 13.6, 13.5. m/z +ve mode – 528.2.

Lumefantrine dodecyl sulfate: [$C_{25}H_{30}I_2NO^+$][$C_{12}H_{25}O_4S^-$]. ^{1}H-NMR (DMSO-*d*$_6$) δ 9.36 (s, 1H), 8.21 (m, 2H), 8.03 (d, *J* = 8.5 Hz, 1H), 7.71-7.54 (m, 5H), 7.52 (dd, *J* = 8.4, 1.8 Hz, 1H), 7.38 (d, *J* = 1.7 Hz, 1H), 6.60 (s, 1H), 5.75 (s, 1H), 3.66 (t, *J* = 6.7 Hz, 2H), 3.30-3.11 (m, 2H), 1.65 (s, 6H), 1.55-1.16 (m, 22H), 1.01-0.76 (m, 9H). ^{13}C-NMR (DMSO-*d*$_6$) δ 141.4, 138.3, 135.1, 134.4, 133.6, 132.6, 131.6, 130.8, 130.7, 129.0, 128.3, 127.1, 125.3, 122.8, 121.2, 65.4, 56.5, 53.6, 51.9, 31.3, 29.1, 29.0, 28.8, 28.7, 25.5, 24.7, 22.1, 19.4, 13.9, 13.5. HRMS (*m*/*z*): [$C_{30}H_{33}Cl_3NO^+$][$C_{12}H_{25}O_4S^-$] requires [$C_{30}H_{33}Cl_3NO^+$] 528.1628 and [$C_{12}H_{25}O_4S^-$] 265.1474; found 528.1626 and 265.1464.

Lumefantrine docusate: [$C_{30}H_{33}Cl_3NO^+$][$C_{20}H_{37}O_7S^-$]. Melting point: 52-60 °C. ^{1}H-NMR (DMSO-*d*$_6$) δ 9.28 (s, 1H), 8.35-8.11 (m, 3H), 8.01 (d, *J* = 8.5 Hz, 1H), 7.71-7.55 (m, 5H), 7.52 (dd, *J* = 8.4, 1.8 Hz, 1H), 7.38 (d, *J* = 1.7 Hz, 1H), 6.61 (d, *J* = 3.7 Hz, 1H), 5.73 (s, 1H), 3.99-3.77 (m, 4H), 3.61 (dd, *J* = 11.5, 3.6 Hz, 1H), 3.30-3.10 (m, 4H), 2.99-2.71 (m, 2H), 1.65 (d, *J* = 6.8 Hz, 4H), 1.49 (s, 2H), 1.42-1.15 (m, 20H), 1.03-0.73 (m, 18H). ^{13}C-NMR (DMSO-*d*$_6$) δ 171.0, 168.3, 141.4, 138.3, 135.1, 134.4, 133.6, 133.5, 132.6, 131.6, 130.8, 130.7, 129.0, 128.3, 127.1, 125.3, 122.8, 121.2, 66.1(2C), 66.0, 61.4,

56.5, 53.6, 51.9, 38.1, 34.1, 29.7, 29.6, 28.3, 23.2, 23.0, 22.4 (2C), 19.4, 13.9 (3C), 13.5, 10.8(3C), 10.7.

HRMS (m/z): [$C_{30}H_{33}Cl_3NO^+$][$C_{20}H_{37}O_7S^-$] requires [$C_{30}H_{33}Cl_3NO^+$] 528.1628 and [$C_{20}H_{37}O_7S^-$] 421.226; found 528.1632 and 421.2246.

Drug Distribution and Aqueous Phase Concentrations for Lumefantrine Free Base and Lumefantrine Docusate in Type I-IV Formulations

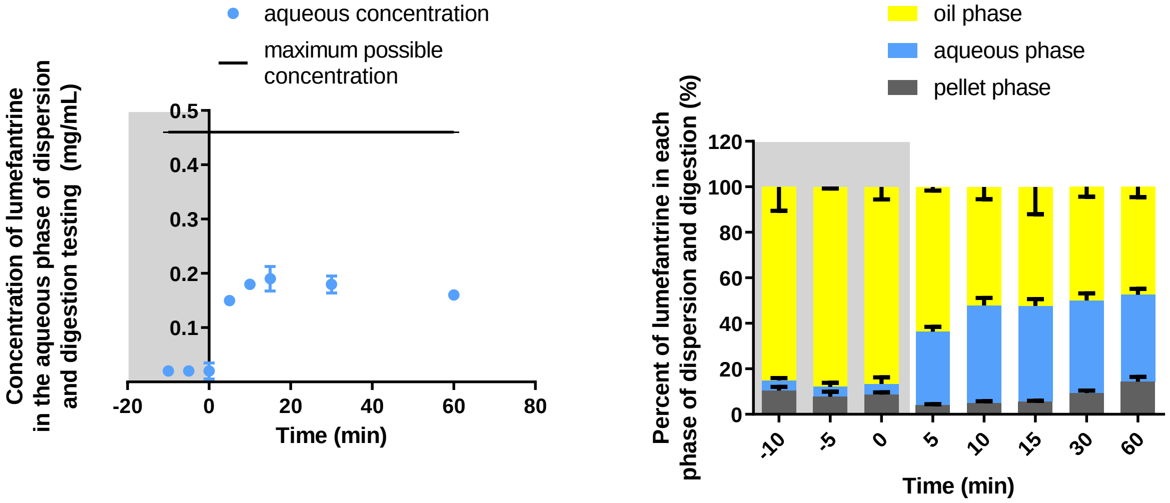

Figure S1. *In vitro* dispersion and digestion of lumefantrine free base in Type I-MCF. The concentration of lumefantrine free base in the aqueous phase of the medium-chain digest as a function of time (left), and the proportion of lumefantrine free base in the pellet, aqueous, and oil phases as a function of time (right). Data are n = 3, mean ± SD.

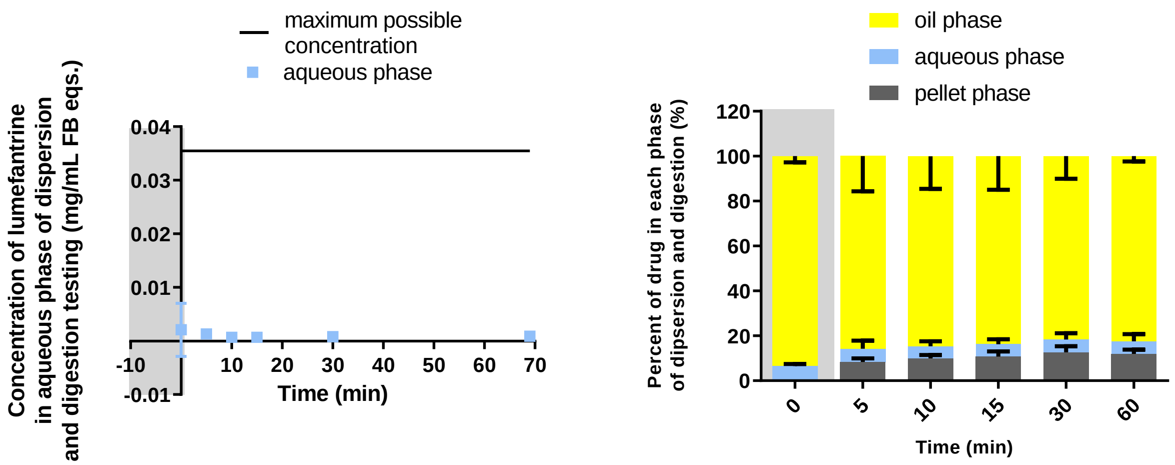

Figure S2. *In vitro* dispersion and digestion of lumefantrine free base in Type I-LCF. The concentration of lumefantrine free base in the aqueous phase of the long-chain digest as a function of time (left), and the proportion of lumefantrine free base in the pellet, aqueous, and oil phases as a function of time (right). Data are n = 3, mean ± SD.

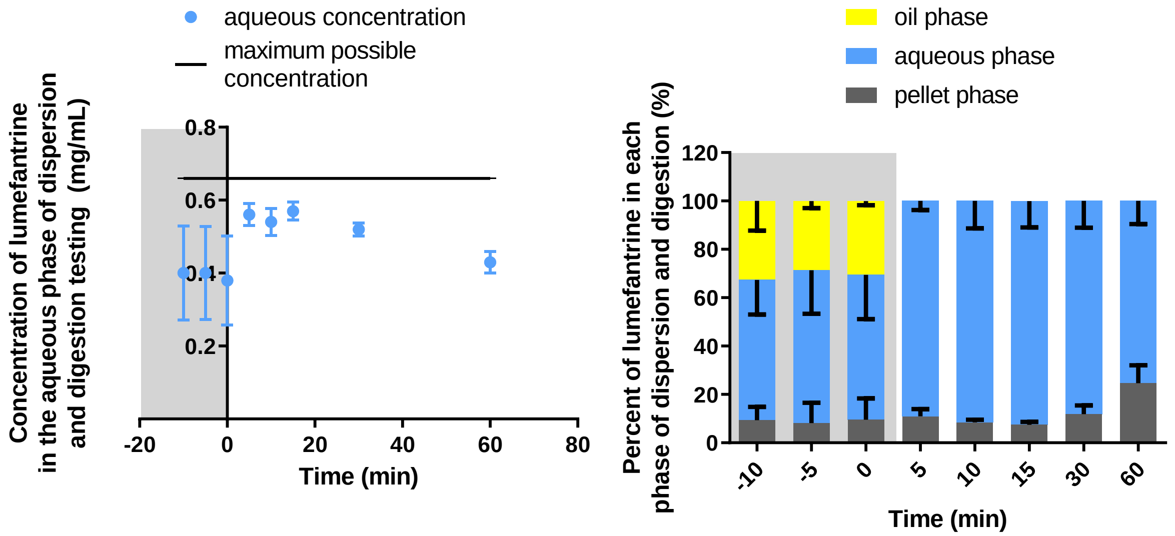

Figure S3. *In vitro* dispersion and digestion of lumefantrine free base in Type II-MCF. The concentration of lumefantrine free base in the aqueous phase of the medium-chain digest as a function of time (left), and the proportion of lumefantrine free base in the pellet, aqueous, and oil phases as a function of time (right). Data are n = 3, mean ± SD.

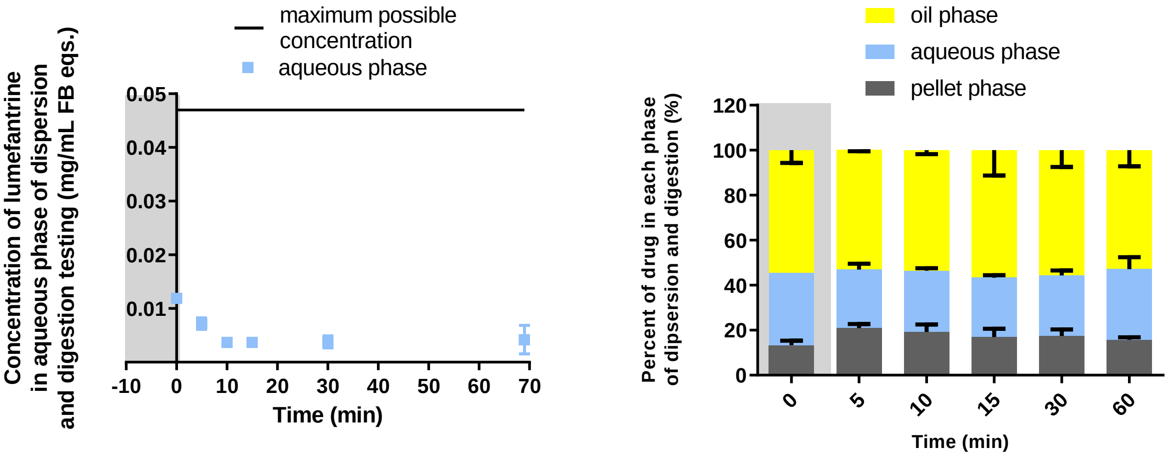

Figure S4. *In vitro* dispersion and digestion of lumefantrine free base in Type II-LCF. The concentration of lumefantrine free base in the aqueous phase of the long-chain digest as a function of time (left), and the proportion of lumefantrine free base in the pellet, aqueous, and oil phases as a function of time (right). Data are n = 3, mean ± SD.

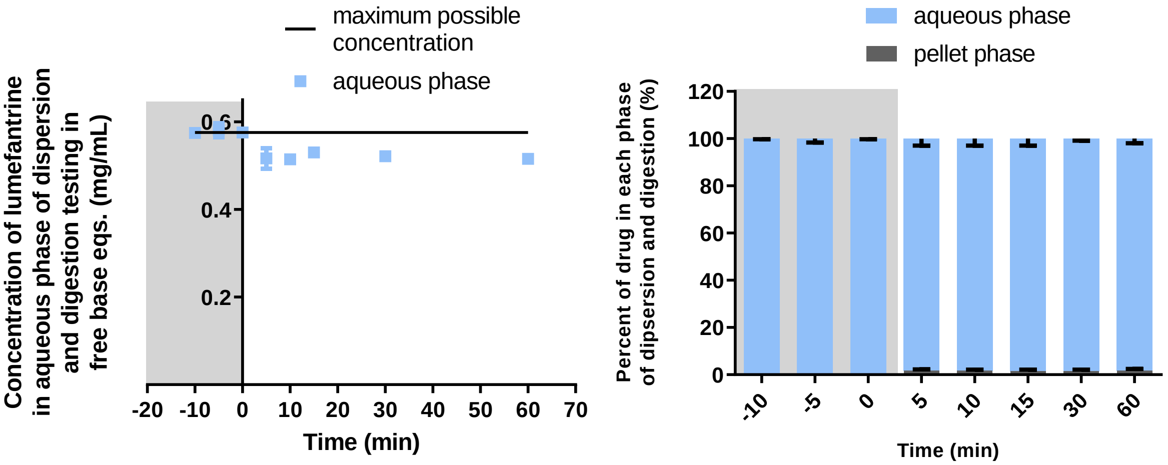

Figure S5. *In vitro* dispersion and digestion of lumefantrine free base in Type IIIA-MCF. The concentration of lumefantrine free base in the aqueous phase of the medium-chain digest as a function of time (left), and the proportion of lumefantrine free base in the pellet and aqueous phases as a function of time (right). Data are n = 3, mean ± SD.

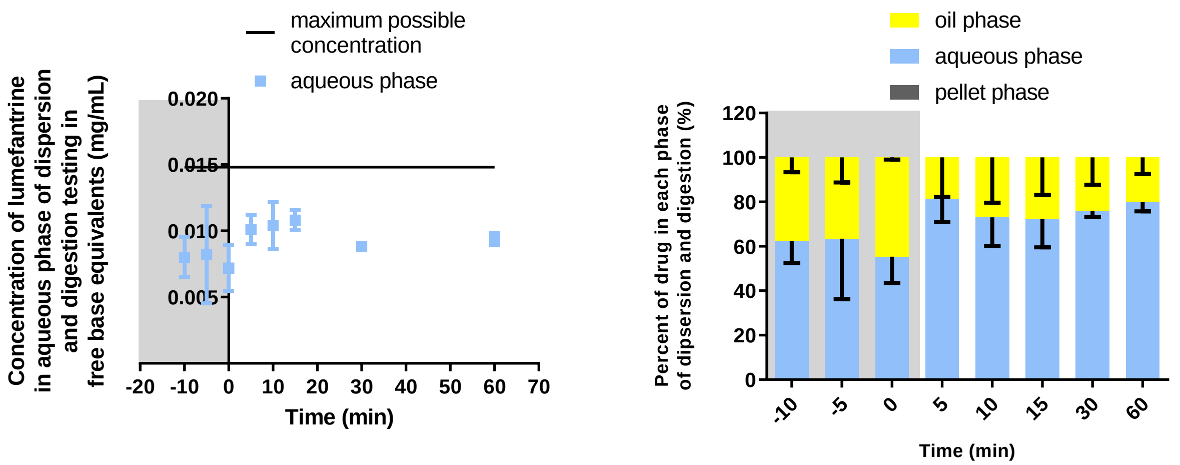

Figure S6. *In vitro* dispersion and digestion of lumefantrine free base in Type IIIA-LCF. The concentration of lumefantrine free base in the aqueous phase of the long-chain digest as a function of time (left), and the proportion of lumefantrine free base in the pellet, aqueous, and oil phases as a function of time (right). Data are n = 3, mean ± SD.

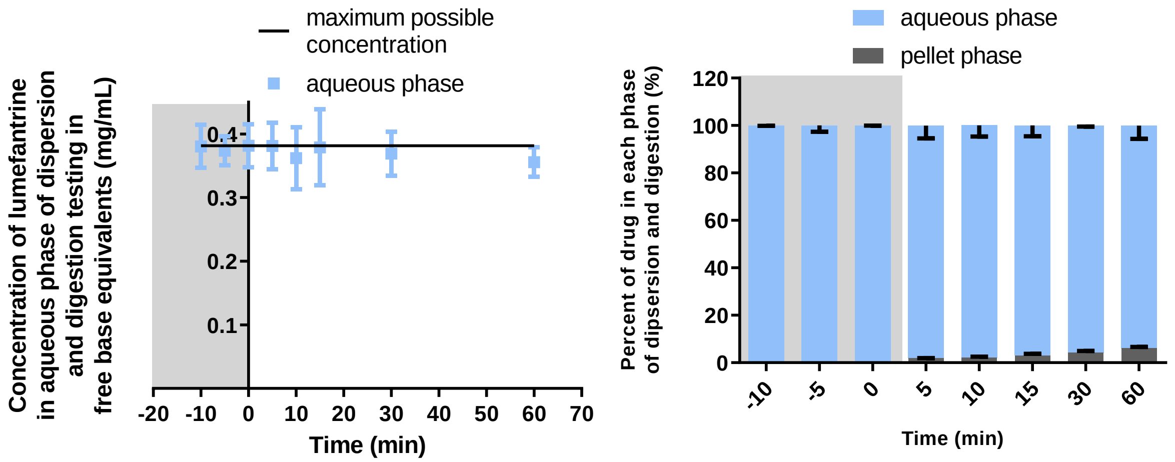

Figure S7. *In vitro* dispersion and digestion of lumefantrine free base in Type IIIB-MCF. The concentration of lumefantrine free base in the aqueous phase of the medium-chain digest as a function of time (left), and the proportion of lumefantrine free base in the pellet and aqueous phases as a function of time (right). Data are n = 3, mean ± SD.

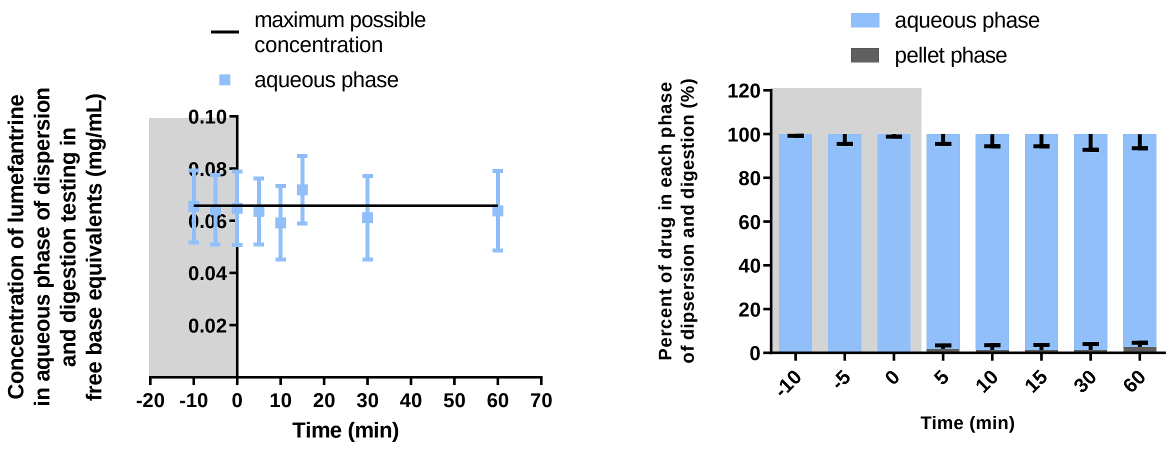

Figure S8. *In vitro* dispersion and digestion of lumefantrine free base in Type IIIB-LCF. The concentration of lumefantrine free base in the aqueous phase of the long-chain digest as a function of time (left), and the proportion of lumefantrine free base in the pellet and aqueous phases as a function of time (right). Data are n = 3, mean ± SD.

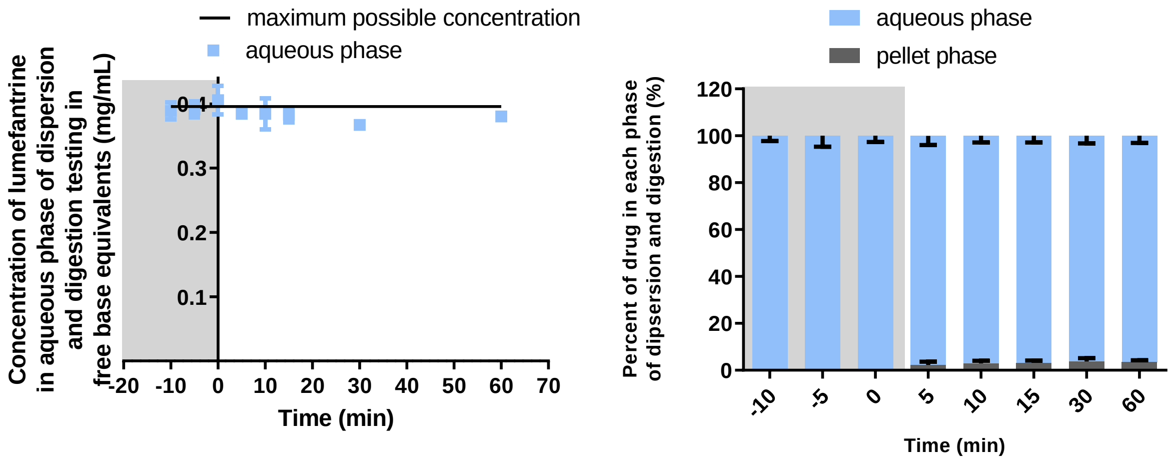

Figure S9. *In vitro* dispersion and digestion of lumefantrine free base in Type IV. The concentration of lumefantrine free base in the aqueous phase of the digest as a function of time (left), and the proportion of lumefantrine free base in the pellet and aqueous phases as a function of time (right). Data are n = 3, mean ± SD.

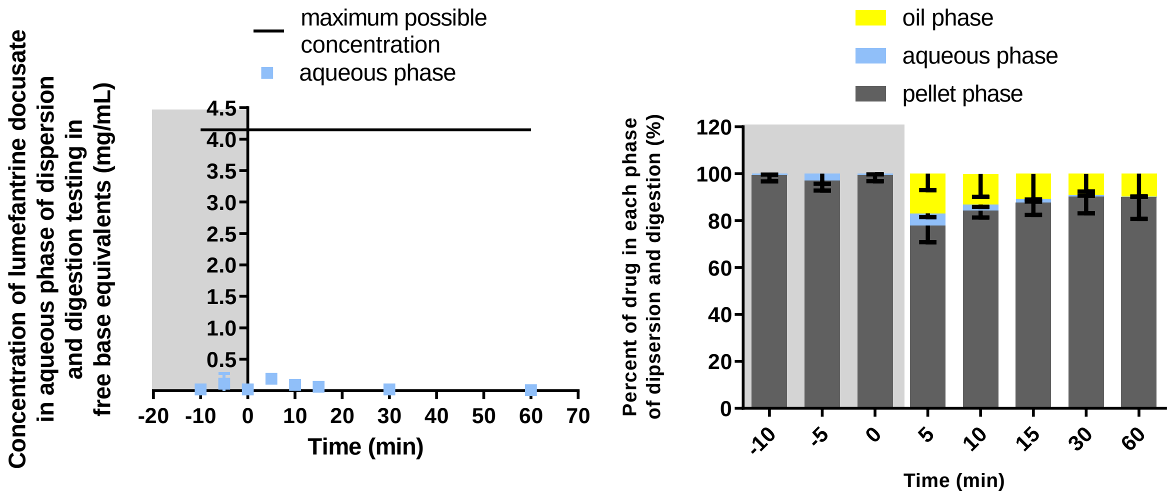

Figure S10. *In vitro* dispersion and digestion of lumefantrine docusate in Type I-MCF. The concentration of lumefantrine docusate (in free base equivalents) in the aqueous phase of the medium-chain digest as a function of time (left), and the proportion of lumefantrine docusate in the pellet, aqueous, and oil phases as a function of time (right). Data are n = 3, mean ± SD.

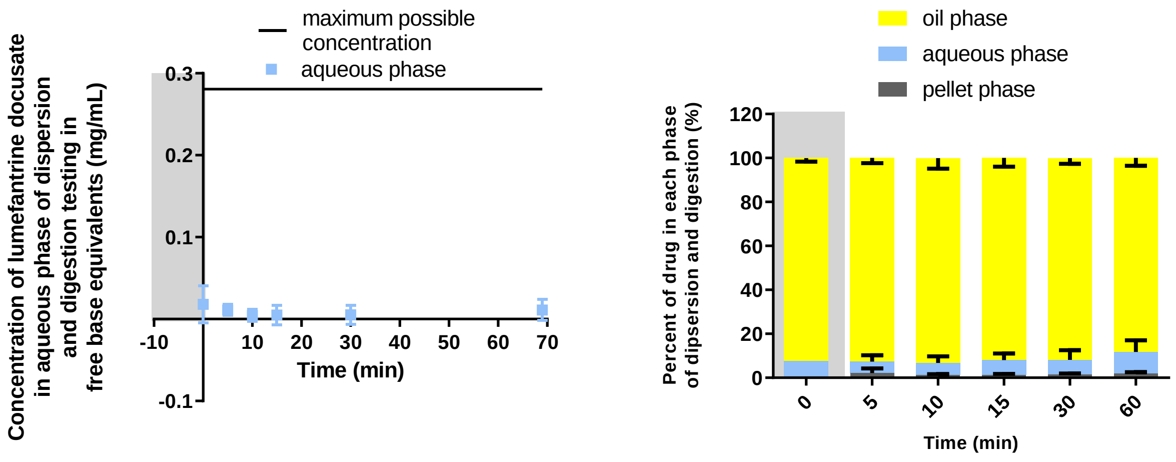

Figure S11. *In vitro* dispersion and digestion of lumefantrine docusate in Type I-LCF. The concentration of lumefantrine docusate (in free base equivalents) in the aqueous phase of the long-chain digest as a function of time (left), and the proportion of lumefantrine docusate in the pellet, aqueous, and oil phases as a function of time (right). Data are n = 3, mean ± SD.

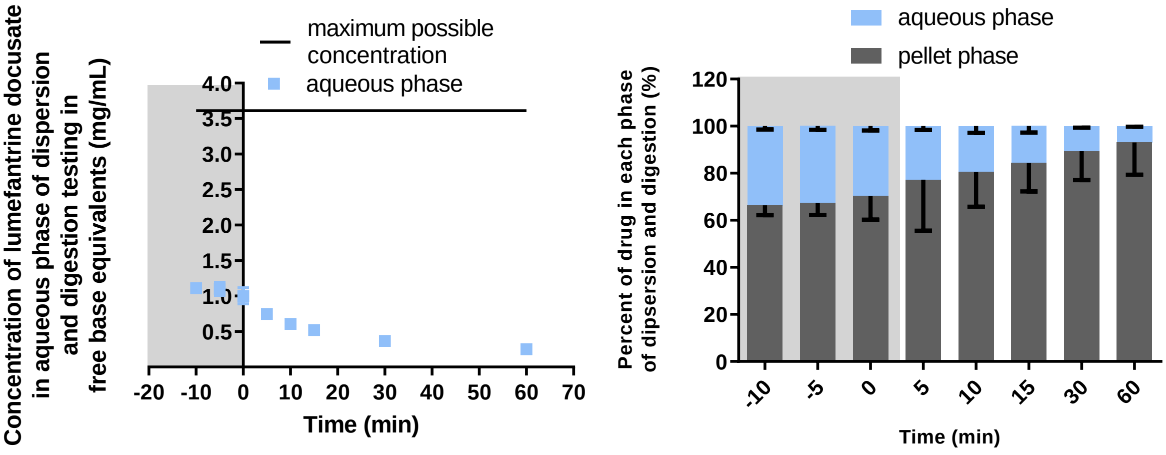

Figure S12. *In vitro* dispersion and digestion of lumefantrine docusate in Type II-MCF. The concentration of lumefantrine docusate (in free base equivalents) in the aqueous phase of the medium-chain digest as a function of time (left), and the proportion of lumefantrine docusate in the pellet and aqueous phases as a function of time (right). Data are n = 3, mean ± SD.

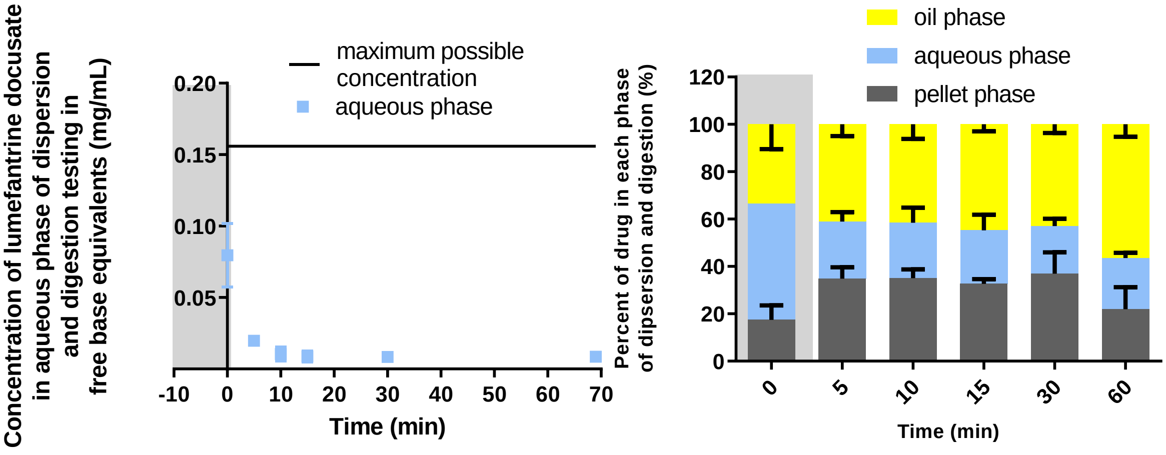

Figure S13. *In vitro* dispersion and digestion of lumefantrine docusate in Type II-LCF. The concentration of lumefantrine docusate (in free base equivalents) in the aqueous phase of the long-chain digest as a function of time (left), and the proportion of lumefantrine docusate in the pellet, aqueous, and oil phases as a function of time (right). Data are n = 3, mean ± SD.

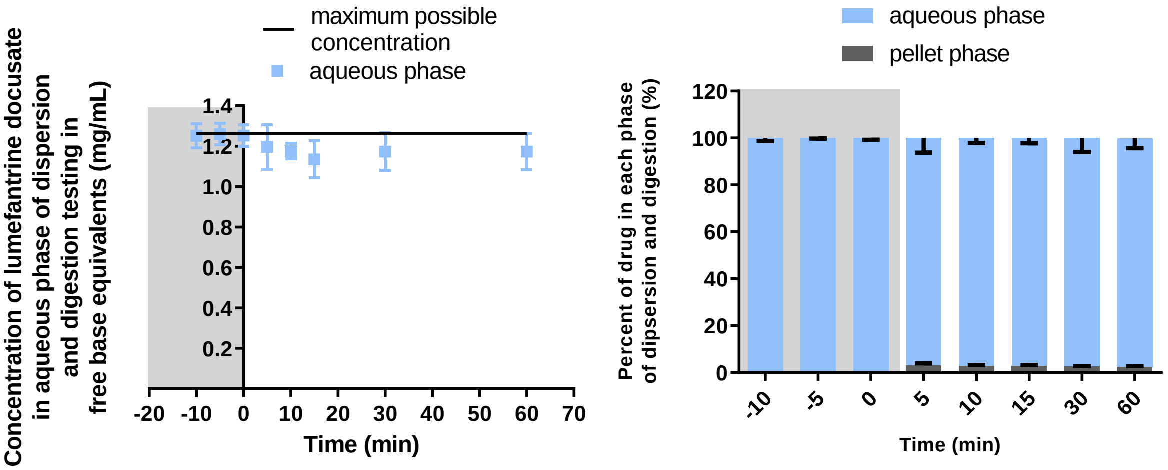

Figure S14. *In vitro* dispersion and digestion of lumefantrine docusate in Type IIIA-MCF. The concentration of lumefantrine docusate (in free base equivalents) in the aqueous phase of the medium-chain digest as a function of time (left), and the proportion of lumefantrine docusate in the pellet and aqueous phases as a function of time (right). Data are n = 3, mean ± SD.

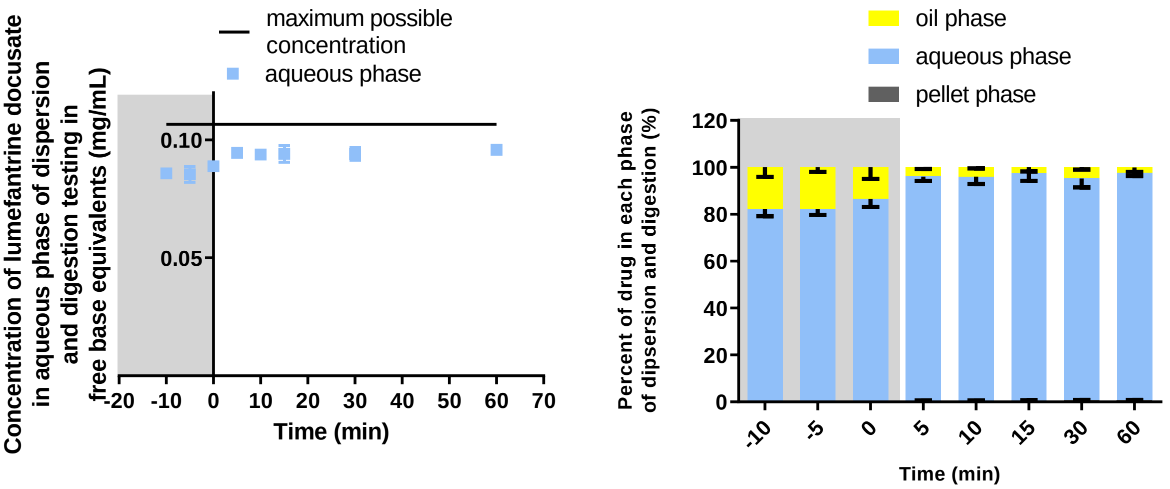

Figure S15. *In vitro* dispersion and digestion of lumefantrine docusate in Type IIIA-LCF. The concentration of lumefantrine docusate (in free base equivalents) in the aqueous phase of the long-chain digest as a function of time (left), and the proportion of lumefantrine docusate in the pellet, aqueous, and oil phases as a function of time (right). Data are n = 3, mean ± SD.

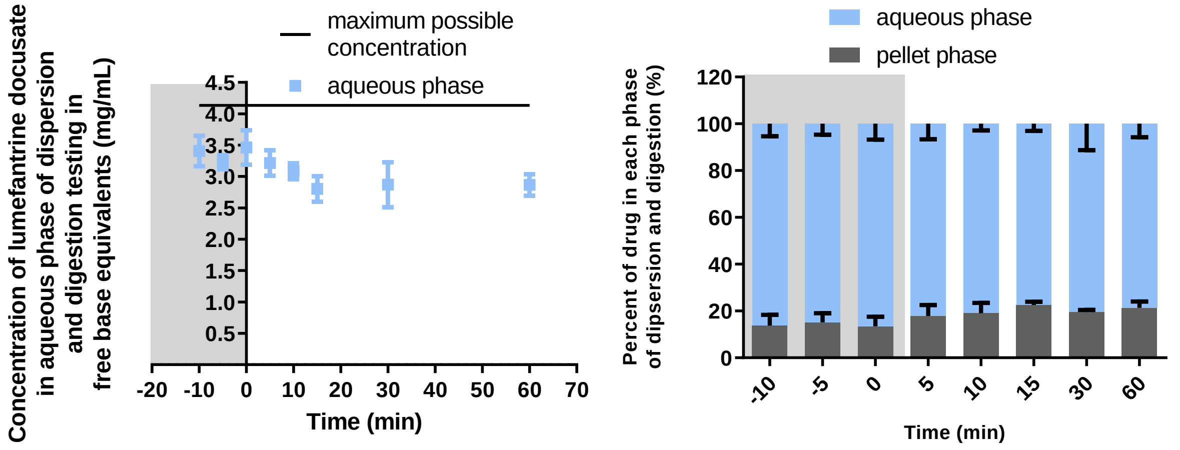

Figure S16. *In vitro* dispersion and digestion of lumefantrine docusate in Type IIIB-MCF. The concentration of lumefantrine docusate (in free base equivalents) in the aqueous phase of the medium-chain digest as a function of time (left), and the proportion of lumefantrine docusate in the pellet and aqueous phases as a function of time (right). Data are n = 3, mean ± SD.

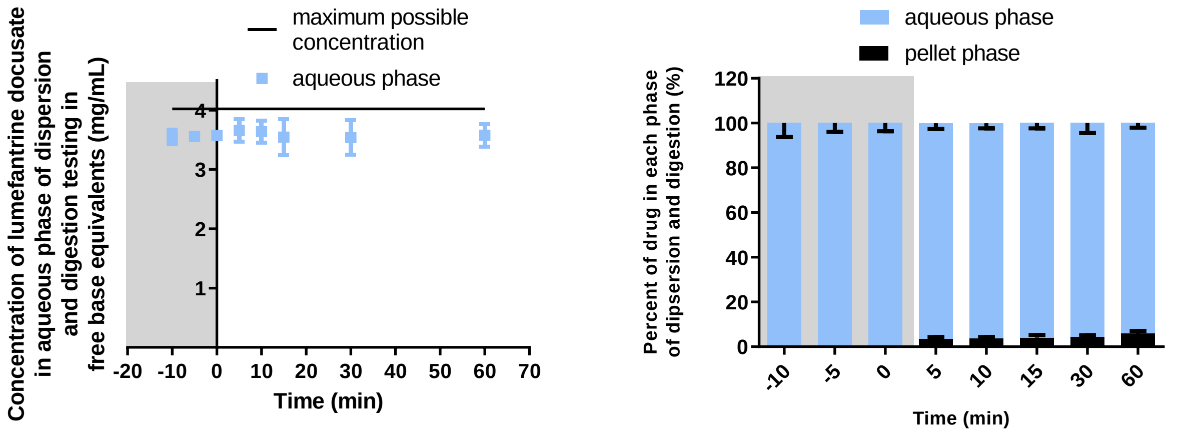

Figure S17. *In vitro* dispersion and digestion of lumefantrine docusate in Type IIIB-LCF. The concentration of lumefantrine docusate (in free base equivalents) in the aqueous phase of the long-chain digest as a function of time (left), and the proportion of lumefantrine docusate in the pellet and aqueous phases as a function of time (right). Data are n = 3, mean ± SD.

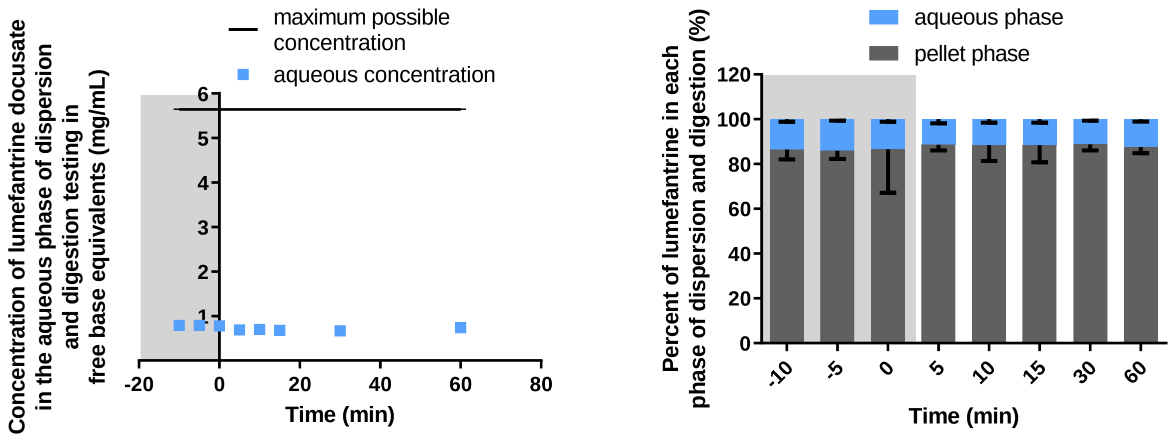

Figure S18. *In vitro* dispersion and digestion of lumefantrine docusate in Type IV. The concentration of lumefantrine docusate (in free base equivalents) in the aqueous phase of the digest as a function of time (left), and the proportion of lumefantrine docusate in the pellet and aqueous phases as a function of time (right). Data are n = 3, mean ± SD.

Dose Normalised (all doses 50 mg/kg) Pharmacokinetic Data

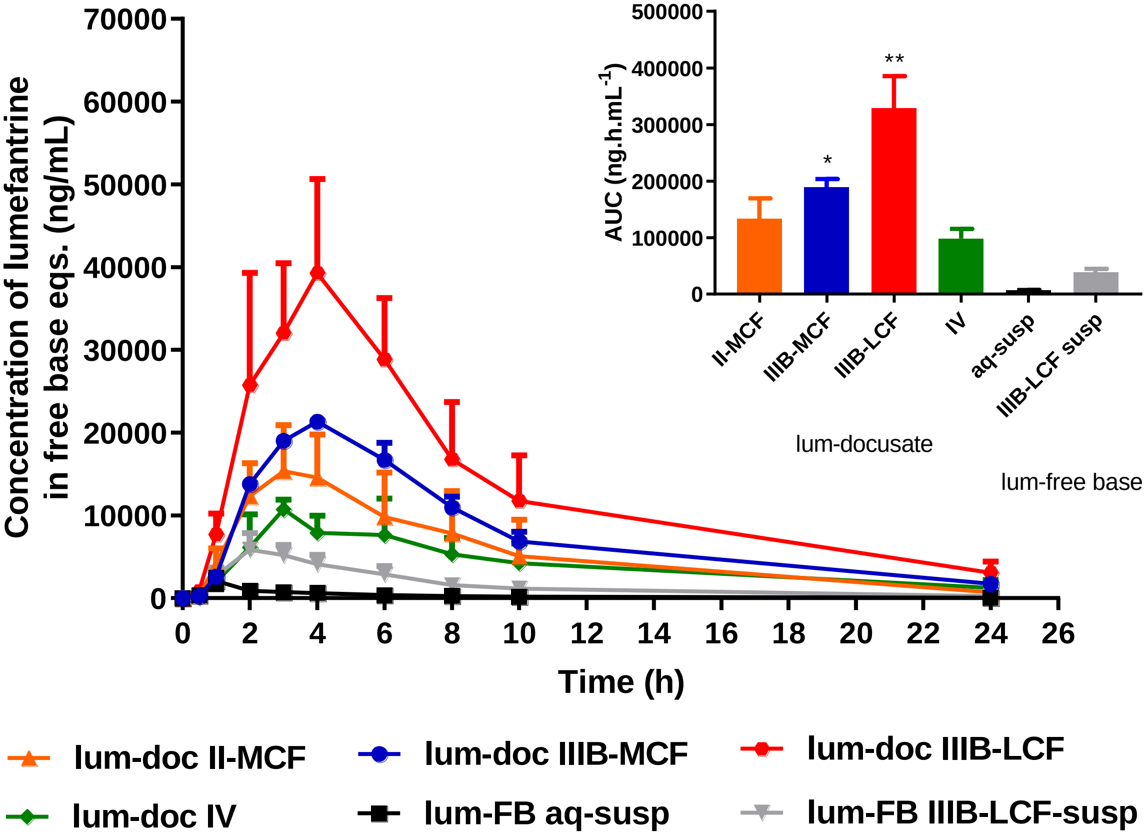

Figure S19. Dose normalized lumefantrine plasma concentration versus time data after oral administration of lumefantrine docusate in Type II-MCF, IIIB-MCF, IIIB-LCF, and IV LBF, as well as lumefantrine free base as an aqueous suspension, and a lipid suspension (in the Type IIIB-LCF). All data have been dose normalized to 50 mg/kg (reflecting the lowest dose administered (Type II-MCF)). Data represented as mean (n = 4) ± SEM. Insert: Total lumefantrine exposure over 24 h. Data represented as mean (n = 4) ± SEM. *Exposure was statistically higher ($p < 0.05$) than both suspension formulations. **Exposure was statistically higher than all other formulations.

In vivo AUC vs. Supersaturation Ratio Correlation Plot

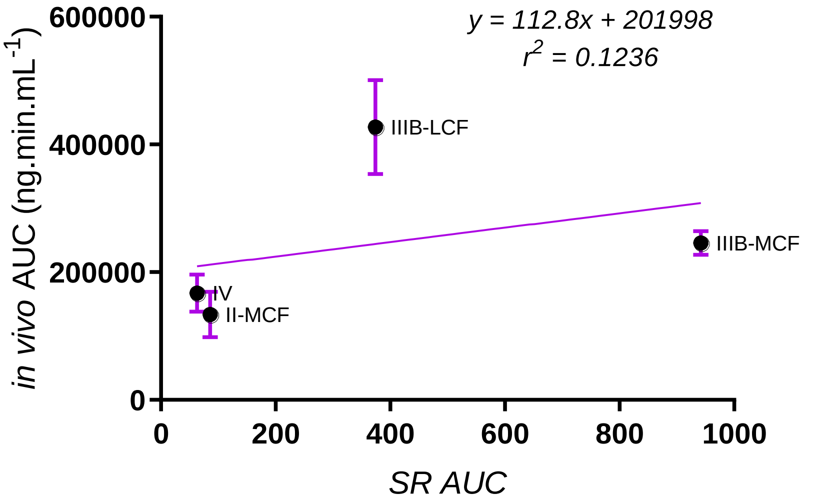

Figure S20. Apparent supersaturation ratio/*in vivo* correlation plot, displaying the AUC of the apparent supersaturation ratio across the *in vitro* experiment, and the AUC for *in vivo* exposure. Data are expressed as mean ± SEM for *in vivo* AUC (n = 4).
